# Cell Death in Liver Disease and Liver Surgery

**DOI:** 10.3390/biomedicines12030559

**Published:** 2024-03-01

**Authors:** Christian Stoess, Yeon-Kyung Choi, Janset Onyuru, Helmut Friess, Hal M. Hoffman, Daniel Hartmann, Ariel E. Feldstein

**Affiliations:** 1 Department of Pediatric Gastroenterology, University of California San Diego, 9500 Gilman Dr, La Jolla, CA 92093, USA; christian.stoess@tum.de (C.S.);; 2 Department of Surgery, TUM School of Medicine, Klinikum rechts der Isar, Technical University of Munich, 81675 Munich, Germany; 3 Department of Internal Medicine, School of Medicine, Kyungpook National University Chilgok Hospital, Kyungpook National University, Daegu 41404, Republic of Korea; 4 Department of Pediatric Allergy, Immunology and Rheumatology, University of California San Diego, La Jolla, CA 92093, USA; 5 Novo Nordisk, Global Drug Discovery, Ørestads Boulevard 108, 2300 Copenhagen, Denmark

**Keywords:** cell death, pyroptosis, apoptosis, ferroptosis, cancer, liver surgery, liver disease

## Abstract

Cell death is crucial for maintaining tissue balance and responding to diseases. However, under pathological conditions, the surge in dying cells results in an overwhelming presence of cell debris and the release of danger signals. In the liver, this gives rise to hepatic inflammation and hepatocellular cell death, which are key factors in various liver diseases caused by viruses, toxins, metabolic issues, or autoimmune factors. Both clinical and in vivo studies strongly affirm that hepatocyte death serves as a catalyst in the progression of liver disease. This advancement is characterized by successive stages of inflammation, fibrosis, and cirrhosis, culminating in a higher risk of tumor development. In this review, we explore pivotal forms of cell death, including apoptosis, pyroptosis, and necroptosis, examining their roles in both acute and chronic liver conditions, including liver cancer. Furthermore, we discuss the significance of cell death in liver surgery and ischemia-reperfusion injury. Our objective is to illuminate the molecular mechanisms governing cell death in liver diseases, as this understanding is crucial for identifying therapeutic opportunities aimed at modulating cell death pathways.

## 1. Introduction

Regulated cell death (RCD) plays an essential role in balancing cell growth and cell turnover within the liver. In the normal physiological state, cell death is non-immunogenic, and even extensive cell death can trigger regenerative, health-restoring responses. In pathogenic cell death, cells work to eliminate threats and initiate immune responses, contributing to the maintenance of organ homeostasis. Nevertheless, a persistent overload of cellular stressors to the liver can disrupt this balance, giving rise to hepatic inflammation and hepatocellular cell death, which are key elements in various liver diseases. Both hepatocytes, as primary liver cells, and non-parenchymal cells, such as Kupffer cells and hepatic stellate cells (HSCs), can undergo different forms of RCD. Such forms include non-lytic apoptosis, as well as lytic necroptosis, pyroptosis, and ferroptosis. Lytic mechanisms can trigger robust inflammatory responses due to cell membrane permeabilization and the release of cellular components. Consequently, immune cells are recruited, and quiescent hepatic HSCs begin transforming into active myofibroblasts.

Hepatocellular death is present in nearly all types of human liver diseases, acting as a highly sensitive and early marker in acute and chronic conditions. Contemporary evidence demonstrates that the initiation of liver diseases is primarily driven by hepatocyte death, leading to inflammation, fibrosis, cirrhosis, and hepatocellular carcinoma (HCC) [1,2]. In acute liver diseases (e.g., drug-induced liver injury, ischemia-reperfusion injury, etc.), fulminant cell death is a fundamental cause of liver failure, whereas chronic hepatocyte death and the associated inflammation contribute to the progression of fibrosis. Ongoing fibrosis can ultimately impair liver function and create a conducive environment for tumor development. In liver tumors, RCD processes influence both pro-tumorigenic and anti-tumorigenic responses within the tumor and its tumor microenvironment (TME). This review summarizes recent advancements in our understanding of the role of cell death in acute and chronic liver diseases. We highlight the molecular significance of cell death in acute drug-induced liver injury (DILI), alcohol-associated and metabolic dysfunction-associated steatotic liver disease (ALD and MASLD), liver cancer, tumor microenvironment, and liver surgery/ischemia-reperfusion injury.

## 2. Types of Cell Death

In this chapter, we offer a general overview of the most significant forms of regulated cell death. Additionally, we direct the reader to the summary provided by the Nomenclature Committee on Cell Death (NCCD) published in 2018, which established the standard for defining each type of cell death [3]. An updated version focusing on apoptosis is also available [4].

### 2.1. Pyroptosis

**Figure 1:**
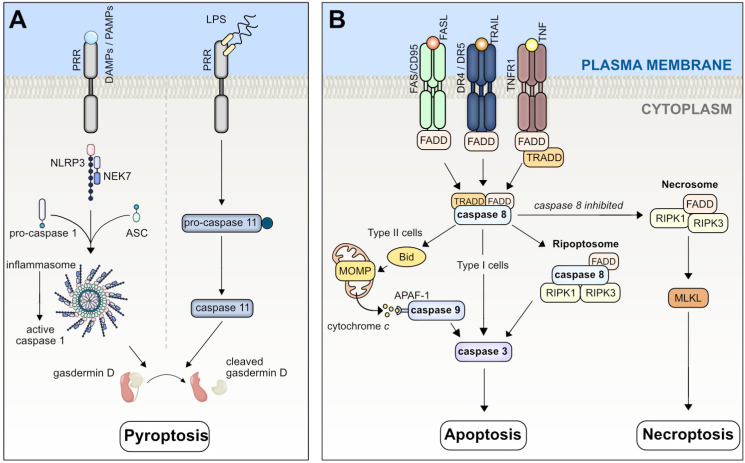
Pyroptosis, apoptosis and necroptosis (simplified graphic). (**A**) Pyroptosis: Pyroptosis is activated via the detection of DAMPs/PAMPs by PRRs. The NLRP3 receptor assembles with NEK7, ASC, and pro-caspase 1 to form the NLRP3 inflammasome, which induces pyroptotic cell death via the cleavage of GSDMD. Alternatively, GSDMD is activated by murine caspase 11 (4/5 in humans). The N-terminal fragments of GSDMD form membrane pores that lead to the release of cell organelles, electrolytes and the pyroptosis-specific cytokines IL-1β and IL-18. (**B**) Apoptosis and necroptosis: Activation of extrinsic apoptotic cell death pathways in response to binding of TNF, TRAIL, and FASL to their respective death receptor culminates in the activation of caspase 8. Cell fate towards survival or cell death is mainly orchestrated through ubiquitylation events that influence the assembly of different scaffolding platforms (e.g., complex IIa: TRADD/FADD/caspase 8). Whereas active complex I (not shown) mainly mediates the activation of the pro-survival NF-κB pathway, different subtypes of complex II mediate the apoptotic cell death when NF-κB is blocked or complex I formation is inhibited. Caspase 8 assembles with TRADD and FADD to form complex IIa and with RIPK1/RIPK3 to form the ripoptosome (complex IIb) which prevents necroptosis and leads to apoptosis by activating caspase 3. When caspase 8 is inhibited, the necrosome (complex IIc) forms and induces lytic necroptosis via MLKL. When MLKL is inhibited, RIPK3 can induce cell death via the activation of pyroptosis. *NIMA-related kinase 7 (NEK7), adaptor molecule apoptosis-associated speck-like protein containing a CARD (ASC), lipopolysaccharide (LPS), apoptotic protease-activating factor 1 (APAF-1)*.

Pyroptosis, as a form of lytic cell death, is observed in chronic liver diseases such as ALD and MASLD/metabolic dysfunction-associated steatohepatitis (MASH) [5]. Pyroptotic cell death leads to the release of proinflammatory cytokines and cellular lysis. This process contributes to immune responses against pathogens but is also implicated in tissue damage in inflammatory diseases [2]. The mechanism is mediated by inflammasomes, which are multiprotein complexes that sense danger signals and activate the inflammatory protease caspase 1 (Figure 1A). Danger signals include pathogen-associated molecular patterns (PAMPs) derived from bacteria, fungi, or viruses, as well as danger-associated molecular patterns (DAMPs) from the host, including intracellular organelles, proteins, DNA, and RNA. Pattern-recognition receptors (PRRs) on innate immune cells and parenchymal cells recognize DAMPs and PAMPs, respectively. As a result, the transcription of nuclear factor kappa B (NF-κB) target genes increases, leading to the upregulation of expression of the NOD-like receptor family, pyrin domain-containing 3 (NLRP3), and the pro-forms of interleukin-1β (IL-1β), and interleukin-18 (IL-18). However, the mechanism by which NLRP3 senses these extracellular perturbations is incompletely understood. Ultimately, downstream signaling leads to the canonical activation of NLRP3 and caspase 1, triggering the cleavage of Gasdermin D (GSDMD). The non-canonical cleavage of GSDMD is carried out by human caspase 4/5, or the murine homologue, caspase 11. These caspases are activated by bacterial lipopolysaccharide (LPS) and do not require the assembly of the NLRP3 inflammasome. After cleavage, the cytotoxic N-terminal fragments of GSDMD assemble to form cell membrane pores, initiating cell death. Additionally, active caspase 1 cleaves immature pro-IL-1β and pro-IL-18, and both cytokines are released via the pores. GSDMD-mediated pore formation is suppressed by the cell-surface protein Ninjurin-1 (NINJ1) [6]. In specific circumstances and within certain cell types, the release of pro-inflammatory cytokines and intracellular DAMPs occurs without triggering cell death [7]. While pyroptosis involves the activation of inflammasomes and the release of pro-inflammatory cytokines, the existence of overlapping pathways with other forms of cell death indicates the presence of a highly intricate and complex control system [8,9].

### 2.2. Apoptosis

Apoptosis, a regulated (non-lytic) form of cell death, plays a vital role in maintaining tissue homeostasis and is heavily involved in the pathologic processes of chronic liver diseases (Figure 1B) [10]. Two well-described pathways regulate the activation of effector caspases 3 and 7, leading to chromatin condensation, proteolysis, and nuclear fragmentation [3]. The extrinsic apoptotic pathway is initiated by extracellular perturbations that are sensed by death receptors (DRs), namely tumor necrosis factor receptor (TNFR), FAS (also known as CD95 or APO-1), or TNF-related, apoptosis-inducing ligand receptor (TRAIL, also known as APO-2). Activation of these receptors leads to the assembly of a scaffold protein called Death-inducing signaling complex (DISC) or complex I, which usually promotes survival. Complex I serves as a scaffold for receptor-interacting protein kinase-1 (RIPK1), a cellular inhibitor of apoptosis 1 and 2 (cIAP1 and 2), TNF receptor-associated factors 2 or 5 (TRAF2 or TRAF5), and the adaptor TNFR-associated death domain (TRADD). After assembling, post-translational modifications, such as ubiquitination, either promote survival or cell death. For instance, deubiquitinated RIPK1 is released from complex I and forms complex IIa with FAS-associated protein with death domain (FADD) and caspase 8 to induce apoptosis in type I cells (where extrinsic apoptosis is independent of mitochondria) and lymphocytes. In type II cells such as hepatocytes, the formation of complex IIa can be blocked by x-linked inhibitor of apoptosis (XIAP). In contrast, when RIPK1 is polyubiquitinated, complex I activates the NFκΒ pathway, which leads to the transcription of pro-survival genes that prevent cell death. In some cell types (type II cells), including hepatocytes, the extrinsic pathway requires the additional cleavage of BH3-interacting domain death agonist (Bid), a pro-apoptotic member of the B-cell lymphoma 2 (BCL-2) protein family, into tBid by caspase 8 for cell death execution (see Figure 1A). In this scenario, tBid translocates into mitochondria and facilitates the release of cytochrome *c*. Following the release of cytochrome *c*, a supramolecular complex known as the apoptosome becomes activated and enhances the activation of protease caspase 9 and effector proteases caspase 3 and 7. The intrinsic apoptotic pathway is commonly activated by intracellular injury such as DNA damage or oxidative stress, which triggers apoptosis via pro-apoptotic members of the BCL-2 family, namely BCL2-associated X, apoptosis regulator (BAX) and/or BCL2 antagonist/killer (BAK) [3]. BAX/BAK facilitates mitochondrial outer membrane permeabilization (MOMP), leading to the release of cytochrome *c*, followed by the activation of caspase 3 and 7. While apoptosis is vital for maintaining cell homeostasis, it serves as the predominant mode of cell death in many liver diseases. Under pathological circumstances, the presence of cellular leakage and inflammation around apoptotic cells can be attributed to inadequate phagocytosis and efferocytosis of cell debris [11]. Furthermore, it is possible that other forms of cell death may co-exist during the development of chronic liver diseases. In some measure, the inflammatory signals emanating from apoptotic cells may be considered advantageous, as they facilitate hepatocyte regeneration and the restoration of liver function following the loss of hepatocytes through apoptosis [11]. The involvement of apoptosis as a primary mode of cell death is described across the entire spectrum of acute and chronic liver diseases and is also extensively described in earlier reviews [1,10].

### 2.3. Necroptosis

Necroptosis is an alternative form of RCD that exhibits characteristics of both apoptosis and necrosis; in particular, it is activated upon stimulation of TNFR by tumor necrosis factor alpha (TNF-α) in the presence of caspase inhibition [12]. Apoptosis and necroptosis share overlapping intracellular cascades, and the transition from apoptosis to necroptosis is contingent upon reduced caspase 8 activity (Figure 1B) [13,14]. Caspase 8 activation within complex IIb (ripoptosome), composed of RIPK1, FADD, caspase 8, and RIPK3, inhibits necroptosis by preventing the activation of RIPK1 and RIPK3, thereby suppressing necroptosis induction [15]. An imbalance between caspase 8 and RIPK3 activities, precipitated by either the inhibition of caspase 8 or the overexpression of RIPK3, promotes the transition of complex IIb (ripoptosome) into complex IIc (necrosome) [16]. This complex promotes the phosphorylation and oligomerization of mixed lineage kinase domain-like (MLKL) protein [17]. Subsequently, MLKL translocates to the inner leaflet of the plasma membrane, thereby promoting membrane permeabilization and inducing cell death [18]. Caspase 8-mediated cleavage of RIPK1 and RIPK3 serves as a checkpoint of necroptotic cell death. In the presence of caspase 8, cells undergoing cell death default to apoptosis. Therefore, inhibiting caspase 8 becomes crucial for inducing necroptosis, and the potential lethality associated with necroptotic signaling underscores its predominant role as a backup cellular defense mechanism triggered when apoptosis is impeded. Nevertheless, this phenomenon raises the question of how necroptosis contributes to the pathogenesis of human diseases in the presence of fully functional caspases. Necroptotic cell death is immunogenic and can trigger excessive inflammation and cell death by activating innate immune cells or promoting other types of cell death, such as pyroptosis [19].

### 2.4. Ferroptosis

**Figure 2:**
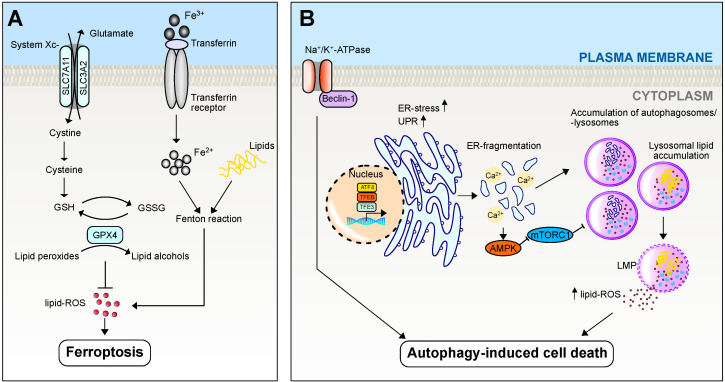
Ferroptosis and autophagy-induced cell death (simplified graphic). (**A**) Ferroptosis: Excessive accumulation of intracellular lipid ROS, coupled with lipid peroxidation, resulting from a reduction in intracellular glutathione (GSH) levels and impaired GPX4 activity, leads to a form of RCD called ferroptosis. (**B**) Autophagy-induced cell death: Autosis, also known as autophagy-dependent cell death, represents a distinct mode of cell demise characterized by specific features arising from imbalanced autophagy or the failure to mitigate cellular stress through regulated autophagy. One notable characteristic of autosis is its susceptibility to inhibition by Na^+^/K^+^-ATPase blockers. Additionally, autophagic cells undergoing autosis can be distinguished by the presence of endoplasmic reticulum (ER) debris. External stressors such as hypoxia or starvation induce the upregulation of autophagy-specific transcription factors and can exacerbate ER stress, triggering the unfolded protein response (UPR) and ultimately leading to ER fragmentation. The ER, acting as a primary reservoir of calcium ions (Ca^2+^), releases Ca^2+^, which in turn activates AMP-activated protein kinase (AMPK). AMPK inhibits the mammalian target of rapamycin complex 1 (mTORC1), a suppressor of autophagy, resulting in the accumulation of autophagosomes and autolysosomes. Furthermore, heightened ER fragmentation fosters lipid accumulation within lysosomes. This lipid buildup culminates in lysosomal membrane permeabilization (LMP), contributing to the generation of lipid-derived reactive oxygen species (ROS). Despite significant progress, the precise processes underlying autosis remain intricate and not yet fully elucidated. *Activating transcription factor 4 (ATF4), transcription factor EB (TFEB), transcription factor E3 (TFE3), solute carrier family 7 member 11 (SLC7A11), solute carrier family 3 member 2 (SLC3A2)*.

Ferroptosis is a novel form of iron-dependent cell death that is morphologically, genetically, and biochemically distinct from other well-known types of cell death [20]. Lipid peroxidation, driven by ferrous iron (Fe^2+^) and free radicals via the Fenton reaction, is a defining feature of ferroptosis (Figure 2A). This process involves the peroxidation of polyunsaturated fatty acids (PUFA) in phospholipids, leading to the generation of PUFA phospholipid hydroperoxides (PUFA-PL-OOH), which can accumulate and potentially cause rapid and irreparable membrane damage [21]. Acyl-CoA synthetase long-chain family member 4 (ACSL4) and lysophosphatidylcholine acyltransferase-3 (LPCAT3) are the pivotal enzymes in the regulation of PUFA biosynthesis and remodeling [22,23]. These enzymes catalyze the conversion of arachidonic acid to PUFA-PL-OOH, which serve as both biomarkers and crucial mediators of ferroptosis [22,23]. When PUFA-PL-OOH cannot be degraded promptly by the glutathione (GSH)-dependent enzyme GPX4, redundant lipid peroxides result in ferroptosis [20,24]. Thus, the inactivation of GPX4 leads to the accumulation of reactive oxygen species (ROS) through lipid peroxidation or through the Fenton reaction via excess iron [24]. Although GPX4 is a major inhibitor of ferroptosis, several GPX4-independent systems, including ferroptosis suppressor protein 1/CoQ10, dihydroorotate dehydrogenase, and GTP cyclohydrolase 1/tetrahydrobiopterin have been recently identified that suppress ferroptosis [25,26,27]. Ferroptosis is also governed by the autophagy-related genes 5 and 7 pathway, along with the nuclear receptor coactivator 4 pathway and the p62/Kelch-like epichlorohydrin-associated protein-1 (KEAP1)/nuclear factor erythroid 2-related factor 2 (NRF2) pathway [28,29]. A significant body of research involving experimental manipulation of ferroptotic processes using specific activators (e.g., RSL3 and erastin) and inhibitors (e.g., ferrostatins) has shed light on the underlying molecular mechanisms of ferroptosis [30]. Ferroptosis has been implicated in the pathogenesis of a diverse range of diseases, including tumors, ischemia-reperfusion injury, neurodegenerative disorders, autoimmune diseases, and hepatic pathologies [31]. Ferroptosis, capable of inducing cell death and impeding tumor progression, can also create an immunosuppressive microenvironment that hampers T-cell activity, ultimately promoting immune evasion by tumor cells [32,33]. Emerging evidence demonstrates intricate crosstalk between tumor cells and immune cells in the context of ferroptosis [34]. Moreover, in the context of liver-related research, the liver is particularly susceptible to oxidative damage, and excessive iron accumulation is a prominent feature in most major liver diseases; therefore, ferroptosis has gained the attention of researchers working in the field of liver disease.

### 2.5. Autophagy/Autophagy-Induced Cell Death

In mammalian cells, three primary forms of autophagy can be distinguished: macroautophagy, microautophagy, and chaperone-mediated autophagy. The complex signaling cascades governing the processes of autophagy are described in detail here [35]. Briefly, the multi-step process is controlled by different kinase complexes, culminating in the degradation and recycling of cellular components in the autophagolysosome [36]. After initiation and elongation, the completion and closure of the autophagosome are mediated by the microtubule-associated protein 1A/1B light chain 3 (MAP1LC3A or LC3) and the endosomal sorting complex required for transport III (ESCRT-III) [37]. Finally, fusion with a lysosome activates the hydrolases and lipases necessary for the degradation of the cellular components. LAMP-1 and LAMP-2, both lysosomal membrane proteins, are required for the fusion process [38]. The concept of autophagy and autophagy-dependent cell death is complex. It is important to differentiate between autophagy, which eliminates damaged organelles to ensure cell survival and three different types of cell death related to autophagy: (1) Autophagy-associated cell death: The induction of autophagy coincides with another form of cell death but does not play an active role; (2) Autophagy-mediated cell death: Autophagy triggers another form of cell death; (3) Autophagy-dependent cell death (also known as ‘autosis’): A form of cell death that depends on the autophagic machinery, involves at least two different autophagy-related proteins, and can be prevented by specific autophagy inhibitors [3]. The multiple features that can induce autophagy-dependent cell death are summarized in Figure 2B. Given the complexity and somewhat artificial boundaries of autophagy and different forms of autophagy-mediated cell death, we will discuss the available data together in the same chapter. The liver, as the main organ regulating the metabolism in the organism, responds to fasting and feeding with basal hepatic autophagy. Thus, autophagy is critical for maintaining the homeostasis and quality control of proteins and organelles in hepatocytes. Autophagy and autophagy-dependent cell death have been described to play a role in metabolic liver diseases such as MASLD/MASH, alcohol-associated liver disease, and drug-induced liver disease, as well as in hepatic tumorigenesis [39].

## 3. Cell Death Responses

Efficiently eliminating apoptotic or necrotic cells is crucial for the maintenance of tissue homeostasis and the prevention of inflammation. This process is intricately governed by several key signals: ‘find me’ signals facilitate phagocyte recruitment; ‘eat me’ signals govern the recognition of dead cells; the absence of ‘don’t eat me’ signals, also known as ‘keep me’ signal in healthy cells, are critical in preventing the inadvertent efferocytosis of viable cells [40]. Collectively, these signals serve as a safeguard, preventing the release of intracellular contents into the surrounding tissues and orchestrating the absorption and degradation of dying cells, thereby playing a pivotal role in maintaining cellular and tissue homeostasis. This significance is underscored by mounting evidence suggesting a correlation between the onset of liver disease and inefficiency in the phagocytic removal of dying cells [41].

### 3.1. ‘Find Me’ Signals

**Figure 3:**
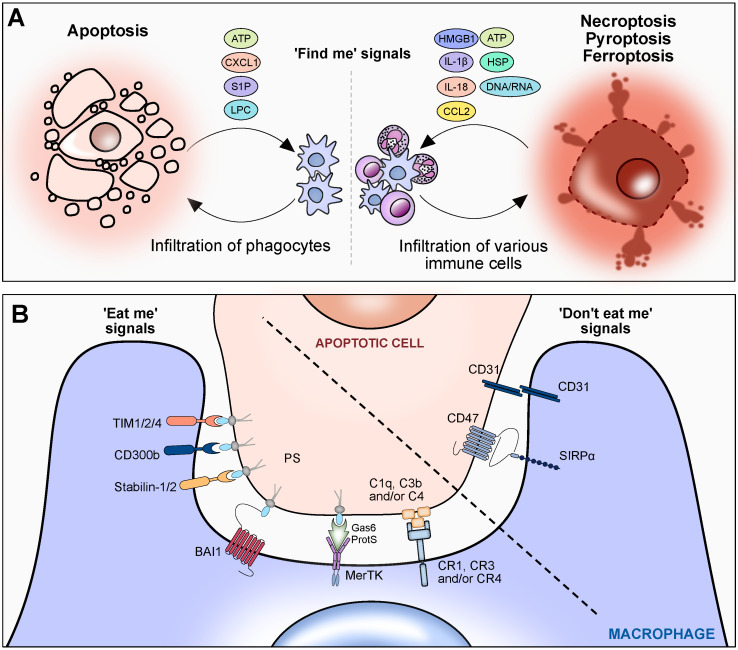
Intercellular regulation of cell death signals (simplified graphic). (**A**) Apoptotic cells release ‘find me’ signals such as sphingosine-1-phosphate (S1P), lysophosphatidylcholine (LPC), C-X-C motif chemokine ligand (CXCL) 1, and nucleotides such as adenosine triphosphate (ATP). S1P and LPC are ‘find me’ signals specifically associated with apoptosis. Phagocytes infiltrate the tissue and remove cell debris. When all debris is phagocytosed, no inflammatory reaction occurs. However, insufficient removal of debris can lead to inflammation and activation of the immune system. Lytic forms of cell death such as pyroptosis, necroptosis, or ferroptosis release pro-inflammatory cytokines as well as DAMPs, which attract various immune cells. (**B**) Phosphatidylserine is the most potent ‘eat-me’ signal of apoptotic cells, which is recognized by macrophage receptors such as CD300b (blue), BAI1 (red), TIM1/2/4 (orange), and Stabilin-1/2 (yellow). The TAM family of receptor kinases Tyro3, Axl, and Mer (gene name Mertk) bind PS indirectly via Gas6 and Protein S. Proteins of the complement cascade, notably C1q and C3b, are expressed by late apoptotic cells and can be recognized by phagocytes. Next to ‘eat me’ signals, cells express also ‘don’t eat me’ signals to negatively regulate phagocytosis. Among these are CD31 and CD47, which binds to signal regulatory protein alpha (SIRPα) on macrophages. *Heat shock protein (HSP), high-mobility group protein B1 (HMGB1), brain-specific angiogenesis inhibitor 1 (BAI1), T-cell immunoglobulin- and mucin-domain-containing molecule (TIM)*.

When cells undergo apoptosis, they release ‘find me’ signals to recruit macrophages and expose ‘eat me’ signals on their surface (Figure 3A,B). To distinguish themselves from healthy cells and attract phagocytes to areas of death, apoptotic cells release ‘find me’ signals such as sphingosine-1-phosphate (S1P), lysophosphatidylcholine (LPC), C-X-C motif chemokine ligand (CXCL) 1 and nucleotides like adenosine triphosphate (ATP) and uridine diphosphate [42]. S1P and LPC are ‘find me’ signals specifically involved in apoptosis. This process involves the upregulation of S1P mitogen-activated protein kinases SPK1 and SPK2 in apoptotic cells, facilitating S1P production from sphingosine and the activation of calcium-independent phospholipase A2 by caspase 3, leading to LPC synthesis from phosphatidylcholine [43,44].

For cells undergoing necroptosis and pyroptosis, the molecular pathway governing efferocytosis is less elucidated compared to apoptosis. However, it is recognized that DAMPs, including nuclear protein high mobility group box-1 (HMGB1), heat shock proteins, purine metabolites like ATP and uric acid, calcium-binding S100 proteins, and inflammatory cytokines IL-1β and IL-18, are produced by necrotic cells [45,46,47,48]. Formyl peptide and CXC chemokines are potent chemoattractants that function as the initial signaling molecules directing neutrophils to areas of focal necrosis in the liver [49,50].

In the context of drug-induced liver injury, CXCL1/CXCL2 signaling through CXCR1/CXCR2 receptors collaborates with formyl peptides to guide neutrophils to necrotic areas [51]. Additionally, CC chemokines such as CCL2 and CCL3 significantly increase in the necrotic liver, and CC chemokine receptor-expressing cells, especially CCR2^+^ monocytes, migrate to the edge of the necrotic area after the initial wave of neutrophil recruitment. This migration, contingent on CCR2 expression, is essential for optimal repair [51,52].

### 3.2. ‘Eat Me’ Signals

Phagocytes, in response to ‘find me’ signals, effectively distinguish and engulf dying cells, displaying ‘eat me’ signals from healthy cells marked by ‘don’t eat me’ signals (Figure 3B). Among the extensively studied ‘eat me’ signals is phosphatidylserine (PS), predominantly located in the inner membrane of healthy cells [53]. ATP11, an ATP-dependent flipase, maintains membrane lipid asymmetry by keeping PS restricted to the inner plasma membrane leaflet. However, the inactivation of ATP11 by caspase 3 during apoptosis facilitates the exposure of PS on the cell surface [54]. PS exposure can also occur in other forms of cell death, though its underlying mechanism remains unclear. Loss of phospholipid asymmetry can result from MLKL activation during necroptosis, intracellular ATP depletion during ferroptosis, or gasdermin-induced pyroptosis [55,56,57].

PS can directly bind to macrophage receptors such as brain-specific angiogenesis inhibitors (BAI), T-cell immunoglobulin- and mucin-domain-containing molecule (TIM) 1/2/4, stabilin-1/ 2, CD300b, and triggering receptor expressed on myeloid cells (TREM) 2 [58]. Alternatively, the interaction between phagocyte surface receptors like MerTK and phosphatidylserine on apoptotic cells is facilitated indirectly through soluble bridging proteins. This interaction is mediated by Protein S and growth arrest-specific 6 (GAS6) dimers, which serve as a bridge between the two entities [59]. Given the pivotal role of dead cell clearance in modulating hepatic inflammation, TIM4 and GAS6 have emerged as crucial elements in the resolution of hepatic-ischemic reperfusion injury [60,61,62]. Furthermore, in chronic liver disease, macrophages expressing stabilin-1, a scavenger receptor, play a role in protecting against liver fibrosis by facilitating the removal of fibrogenic products resulting from lipid peroxidation [63]. An additional distinctive ‘eat me’ signal involves mannose-binding lectin, C1q, C3b, and C4, as well as opsonization by IgG/IgM, resulting in binding to cells expressing integrin CD91/calreticulin, CD11b/CD18, and Fc-gamma receptors [58].

### 3.3. ‘Don’t Eat Me’ Signals

Effective engulfment of dead cells necessitates both the exposure of ‘eat me’ determinants and a concomitant reduction in ‘don’t eat me’ signals on the surface, which are essentially ‘keep me’ signals emitted by healthy cells to inhibit efferocytosis (Figure 3B) [64]. Viable cells express ‘don’t eat me’ signals, including CD31, CD47, CD46, and CD61 [65,66,67]. The downregulation of ‘don’t eat me’ signals, such as CD47 and its binding partner signal regulatory protein α (SIRPα), contributes to the internalization of apoptotic bodies, suggesting a coordinated effort between the dying cell and the phagocyte [65]. In fibrotic diseases like autoimmune fibrosis, the accumulation of CD47 in fibrogenic cells impedes the elimination of diseased fibroblasts [68]. Both human and mouse MASH livers show the accumulation of necroptotic hepatocytes associated with an upregulation of CD47. Treatment with an anti-CD47 antibody not only enhances the uptake of these cells by liver macrophages but also inhibits fibrosis [69]. Plasminogen activator inhibitor-1 (PAI-1) in both plasma and tissues serves as another known ‘don’t eat me’ signal in both viable and apoptotic neutrophils. PAI-1 can inhibit the engulfment of apoptotic neutrophils by macrophages [70].

## 4. Cell Death in Liver Disease

### 4.1. Acetaminophen-Induced Liver Injury

Drug-induced liver injury or DILI is a significant cause of acute liver injury (ALI) that can ultimately progress to acute liver failure (ALF) [71]. The most common cause of intrinsic or direct DILI is acetaminophen (APAP, also known as paracetamol) toxicity, which induces direct, dose-dependent cell death of hepatocytes through mitochondrial damage. APAP-induced hepatocyte death primarily starts in the centrilobular region (zone III) of the liver. Around 50% of ALF cases in the US and Europe can be attributed to APAP overdose [71,72,73]. Direct DILI increases ROS levels, mitochondrial dysfunction, and endoplasmic reticulum (ER) stress, which can eventually lead to cell death [74,75]. APAP is metabolized by the cytochrome P450 enzymes to a reactive metabolite known as N-acetyl-p-benzoquinone imine (NAPQI), which is detoxified by glutathione (GSH). Once GSH is depleted, NAPQI can bind to cellular proteins, causing mitochondrial damage and ROS production, eventually leading to many types of cell death discussed below [76].

#### 4.1.1. Pyroptosis

The role of pyroptosis in APAP-induced ALI is controversial. In a carbon tetrachloride (CCl_4_)-induced ALI model, the treatment with the NLRP3-specific inhibitor MCC950 mitigated tissue damage by enhancing M2 macrophage polarization and myeloid-derived suppressor cell function at various time points of ALI [77]. However, *Gsdmd* and *Gsdme* knockout (KO) mice did not exhibit any improvement of liver injury in the APAP or thioacetamide (TAA) model at a 24-h time point [78]. In another study, *Gsdmd*^−/−^ mice had significantly increased liver damage at 6 h after APAP compared to wild-type (WT) mice [79]. The authors attributed the escalation in injury, resulting from the inhibition of GSDMD-mediated pyroptosis, to increased levels of apoptosis and necroptosis. Nevertheless, the absence or inhibition of caspase and MLKL, respectively, does not exhibit any impact on cell death induced by APAP. One plausible explanation for the differing study results could be related to the use of a different substrain of WT control mice, as discussed in a review by Shojaie et al. [10]. In summary, inhibiting pyroptosis and deleting GSDMD/GSDME do not appear to prevent APAP-induced liver cell death and might even accelerate it. However, the intricate mechanisms involved in DILI underscore the need for more studies, especially with time-course relevant experiments, to delineate the impact of cell death on disease progression and resolution.

#### 4.1.2. Apoptosis

The reactive metabolite NAPQI leads to mitochondrial dysfunction and nuclear DNA fragmentation, all key features of apoptotic cells. However, current evidence suggests that apoptosis might play a very limited role. For instance, caspase 3 activity and cleaved caspase 3 were not detectable in the plasma of overdosed patients or mice but were elevated after TNF-induced apoptosis, indicating that APAP-hepatotoxicity does not substantially contribute to apoptosis [80]. Similar results were obtained from an in vitro study using an immortalized human hepatocyte cell line, where APAP treatment did not increase caspase activation and treatment with a pan-caspase inhibitor did not protect against APAP-induced cell death. In contrast, TNF-induced apoptosis increased the caspase activity in these cells [81]. However, data from another study suggest that caspase activation and apoptosis are involved in the case of APAP-overdosed patients with spontaneous recovery, whereas caspase-independent cell death might be more relevant in irreversible forms of liver failure [82]. In human hepatocytes, treatment with APAP leads to GSH depletion, the creation of protein adducts, and mitochondrial dysfunction resembling APAP overdose in vivo. Furthermore, APAP treatment results in time-dependent c-Jun N-terminal kinase (JNK) activation in the cytosol, a pathway known to be involved in apoptotic cell death [83,84]. In summary, under certain experimental conditions, features of apoptosis might be detectable in the setting of APAP. However, considering the absence of caspase activity and the ineffectiveness of caspase inhibition in preventing APAP-induced damage, it appears that the contribution of apoptosis to APAP toxicity may be insignificant.

#### 4.1.3. Necroptosis

According to available data, APAP-related liver injury is not substantially impacted by the necroptosis signaling pathway, where the necrosome (RIPK1-RIPK3-MLKL) acts as the key mediator of necroptotic cell death [75]. Moreover, independently of necroptosis, RIPK1 has been shown to signal by means of c-Jun NH2-terminal kinase, contributing to the execution of necrotic cell death. Nevertheless, the role of RIPK1 remains a subject of controversy, as an antisense oligonucleotide approach against RIPK1 demonstrated amelioration of acetaminophen-induced cell death [75], while both full-body and hepatocyte-specific *Ripk1*^−/−^ mice showed either harmful or negligible effects [85,86]. In a separate study, it was shown that the knockdown of RIPK3 and MLKL did not impact the outcome of ALI [78]. However, the chemical inhibition of RIPK1 with necrostatin-1 showed a protective effect against cell death in ALI. The authors concluded that the tumor necrosis factor-like weak inducer of apoptosis (TWEAK)/Tnfrsf12a axis induces excessive apoptosis with RIPK1. In summary, additional data is required to comprehensively understand the role of necroptosis in APAP-induced ALI.

#### 4.1.4. Ferroptosis

Ferroptosis can occur either through direct inhibition of GPX4 or indirectly via the depletion of GSH. This results in the excessive accumulation of lipid peroxides catalyzed by intracellular bioactive iron. In ferroptosis, dysfunction of GPX4 leads to an increase in lipid peroxides derived from polyunsaturated fatty acid-containing phospholipids. Treatment of mice with ferrostatin-1, a specific inhibitor of ferroptosis, reduced the necrotic centrilobular areas and liver enzyme levels, and improved survival. These findings were validated in ACSL4–/Y mice lacking expression of ACSL4, a key enzyme in ferroptosis, and by CRISPR/Cas9-mediated ACSL4 deletion in the liver [87]. Since the depletion of GSH in the setting of APAP-induced liver injury is well known, it appears likely that ferroptosis is indirectly activated via GSH depletion. Primary mouse hepatocytes that were treated with acetaminophen showed improved viability when ferrostatin-1 was added to the media [88]. Targeting ferroptosis via biocompatible poly(acrylic) acid-coated Mn_3_O_4_ nanoparticles reduced ROS production and ferroptotic cell death in an APAP-mouse model [89]. Ferroptosis may be relevant in murine APAP-induced liver injury, but there is limited information available regarding its relevance in humans.

#### 4.1.5. Autophagy/Autophagy-Induced Cell Death

Autophagy serves as a selective mechanism for removing damaged organelles, such as mitochondria, playing a crucial role in safeguarding cells against cell death induced by mitochondrial damage. Hence, autophagy can act as a protective mechanism in APAP overdose. Indeed, APAP treatment induces autophagy both in the mouse liver and in cultured primary hepatocytes. Blocking autophagy with chloroquine intensified the hepatotoxic effects triggered by APAP, while stimulation of autophagy with rapamycin suppressed APAP-induced liver damage [90]. Upon exposure to potential stressors such as APAP, the ER initiates the unfolded protein response (UPR) to restore homeostasis [91]. ER stress is associated with APAP overdose, and upregulation of UPR-related pathways is seen in patients who were liver transplanted because of drug-induced ALF. Among the sensor protein-transcription factors of UPR is X-box binding protein 1, which is upregulated in APAP-overdosed human livers [92]. Recent studies showed how ER stress in APAP-induced liver injury is linked to the upregulation of autophagy to prevent excessive cell stress and death. Therefore, autophagy may have a protective role in APAP-induced liver injury.

### 4.2. Alcohol-Associated Liver Disease

Alcohol-associated liver disease (ALD) encompasses a spectrum from mild to advanced conditions, posing a significant global health concern with outcomes ranging from alcohol-associated hepatitis to cirrhosis. The disease progression is marked by steatosis, alcohol-associated hepatitis, and fibrosis [93]. Accumulating evidence indicates that various programmed cell death pathways, including apoptosis, necroptosis, pyroptosis, and ferroptosis, are critical for the progression of ALD, where intricate interactions between hepatocytes and immune cells play a pivotal role [94].

#### 4.2.1. Pyroptosis

Existing evidence strongly supports a key role of pyroptosis in the pathogenesis of ALD, both in human subjects and animal models [95]. The NLRP3 inflammasome pathway in hepatocytes is activated in response to alcohol, and a deficiency in *Nlrp3* mitigates liver steatosis and injury caused by chronic ethanol exposure [96]. The sterile danger signals, uric acid and ATP, released by damaged hepatocytes, initiate the release of IL-1β in immune cells. Uric acid and ATP serve as secondary DAMPs in the inflammasomal and pyroptotic activation in ALD [97]. Ethanol triggers pyroptosis through the downregulation of microRNA-148a, leading to thioredoxin-interacting protein (TXNIP) overexpression and subsequent NLRP3 inflammasome activation [98]. Additionally, gut-derived PAMPs or metabolic-derived DAMPs induce pyroptosis, resulting in the release of inflammasome-dependent cytokines by immune cells [99,100]. Intriguingly, pyroptosis, mediated through non-canonical caspase-11-dependent cleavage of GSDMD, has been identified as a crucial mechanism in the transition from chronic ALD to alcoholic hepatitis [95]. Pyroptosis activation in both hepatocytes and liver immune cells exacerbates liver inflammation through intercellular crosstalk. A recent study revealed that alcohol consumption induces pyroptosis in Kupffer cells, which release more IL-1β through active m6A enzyme methyltransferase-like 3 [101].

#### 4.2.2. Apoptosis

The metabolic process of ethanol oxidation, involving alcohol dehydrogenase, acetaldehyde dehydrogenase, catalase, and cytochrome P450 2E1, leads to the generation of ROS [102]. This heightened oxygen consumption induces a hypoxic state in hepatocytes, further amplifying ROS production [103]. Chronic alcohol intake triggers excessive ROS production, oxidative stress, and protein adduct formation, disrupting ER protein folding and triggering both intrinsic and extrinsic apoptotic pathways [104]. ROS generation leads to mitochondrial dysfunction and triggers the onset of mitochondrial outer membrane permeability, leading to the release of cytochrome *c*, which in turn promotes the activation of caspase 9 and caspase 3 [105]. Accumulating evidence indicates that Kupffer cells play a pivotal role in the pathogenesis of both chronic and acute ALD [106]. Moreover, alcohol exposure facilitates the hepatic translocation of gut-derived endotoxin/lipopolysaccharide, a potent inducer of M1 polarization in Kupffer cells [107,108]. Consequently, these activated Kupffer cells produce significant amounts of ROS, pro-inflammatory cytokines, and chemokines, which not only lead to liver injury but also stimulate the production of extracellular matrix proteins, thereby inducing characteristic fibrosis [109]. Increased intestinal permeability to macromolecules and endotoxemia has been validated in patients at various stages of ALD characterized by chronic alcohol abuse [110].

#### 4.2.3. Necroptosis

Since inhibiting apoptosis does not prevent ethanol-induced steatosis and inflammation, non-apoptotic cell death pathways, such as necroptosis, gain significance [111,112]. Several investigations have indicated that necroptosis of hepatocytes plays a pivotal role in exacerbating inflammation in ALD. Increased RIPK3 expression in the liver correlates with a poor prognosis post-diagnosis, observed both in murine models and in patients with ALD [113]. Moreover, levels of RIPK1 and RIPK3 in plasma, rather than MLKL, have been identified as potential biomarkers for differentiating alcohol-associated hepatitis from non-alcohol-associated hepatitis. Similarly, *Ripk3*^−/−^ mice exhibit reduced ethanol-induced liver injury, although recent studies question the protective role of MLKL deletion [112]. Notably, myeloid MLKL may perform a non-necroptotic role, modulating macrophage phagocytosis and mitigating ethanol-induced liver inflammation and injury [114]. The exact mechanisms by which necroptosis exacerbates ALD remain under investigation and it is poorly understood how necroptosis in different cell types affects the disease course.

#### 4.2.4. Ferroptosis

Targeting ferroptosis emerges as a potential treatment strategy for ALD. Recent studies have shown that alcohol exposure leads to the accumulation of lipid peroxides and increased mRNA expression of cyclooxygenase-2 while decreasing the protein levels of xCT/SLC7A11 and GPX4 [115]. Ferrostatin-1 was found to markedly mitigate liver damage induced by excessive alcohol consumption in both in vitro and in vivo models [115]. Dimethyl fumarate exhibits a protective effect on ethanol-induced liver injury by activating the NRF2 pathway and inhibiting ROS-induced lipid peroxidation and ferroptosis [116]. Ablation of intestinal sirtuins, a family of signaling proteins involved in metabolic regulation, protects against ethanol-induced liver injury by increasing hepatic GSH levels and regulating iron metabolism and lipid peroxidation [117]. In contrast, adipose-specific overexpression of lipin-1, a Mg^2+^-dependent phosphatidic acid phosphohydrolase, exacerbates alcoholic steatohepatitis, hepatobiliary damage, and fibrogenic reactions by facilitating the onset of hepatic ferroptosis independently of GPX4 [118].

#### 4.2.5. Autophagy/Autophagy-Induced Cell Death

Upon ethanol exposure, autophagy protects the liver in both hepatocytes and Kupffer cells by eliminating misfolded proteins and reducing lipid accumulation [119]. Hence, impairments in lysosomal function and autophagy are key factors in the development of ALD. Chronic alcohol exposure attenuates autophagy through several pathways, including the suppression of AMPK activity and disruption of vesicular movement within hepatocytes [120]. The decline of autophagy leads to the accumulation of damaged organelles in hepatocytes. Studies have demonstrated that acute ethanol intake increases nuclear transcription factor EB (TFEB) levels, a master regulator of lysosomal biogenesis and autophagy, whereas chronic exposure results in reduced nuclear TFEB in both animals and humans [121,122]. The dysfunction of autophagic processes is associated with the pathological effects of alcohol consumption, including protein aggregate formation and mitochondrial damage. Furthermore, the inability to eliminate damaged mitochondria through autophagy can lead to the disruption of oxidative phosphorylation and cell death, highlighting the crucial role of autophagy in preserving liver cell viability against alcohol-induced harm.

### 4.3. Metabolic Dysfunction-Associated Steatotic Liver Disease

Metabolic dysfunction-associated steatotic liver disease (MASLD) is the most prevalent liver disorder globally, affecting 25% of the population [100]. Within this context, studies indicate that 20% of MASLD patients progress to metabolic dysfunction-associated steatohepatitis (MASH), marked by steatosis accompanied by ballooning hepatocytes, the presence of Mallory bodies, and inflammatory processes that culminate in fibrosis [123]. There is strong evidence that multiple forms of hepatocyte cell death drive MASH-associated inflammation and fibrosis, as discussed below.

#### 4.3.1. Pyroptosis

The detection of mitochondria-released DAMPs, including mitochondrial DNA, mitochondrial ROS, and ATP, can directly or indirectly promote NLRP3 inflammasome activation. Pro-inflammatory cytokines released from pyroptotic hepatocytes and macrophages serve as key molecules in the development and progression of MASLD [124,125]. Gaul et al. demonstrated that hepatocytes undergoing NLRP3-mediated pyroptosis amplify inflammasome-driven hepatic fibrogenesis through the engulfment of extracellular NLRP3 inflammasome particles by HSCs [126]. GSDMD, serving as the executor of pyroptosis through its role in forming pores in the cell membrane, plays a fundamental role in triggering the release of pro-inflammatory cytokines. This leads to the involvement of the NF-κB signaling pathway and subsequent recruitment of macrophages in MASH [127]. Moreover, a recent study focusing on the cell-dependent effects of pyroptosis has highlighted pyroptotic interactions between myeloid and hepatic stellate cells. Deletion of *Nlrp3* in myeloid-derived cells resulted in reduced activation of HSCs and liver fibrosis, whereas deletion of *Nlrp3* in HSCs or hepatocytes did not confer protection [128]. Taken together, pyroptosis is identified as a key cell death pathway in MASLD and can drive disease progression to MASH.

#### 4.3.2. Apoptosis

Accumulating evidence suggests that both the intrinsic and extrinsic apoptosis pathways are the prevailing mode of cell death in MASLD [129]. Excessive accumulation of saturated fatty acids in the liver triggers apoptosis, primarily through oxidative and ER stress mechanisms. This accumulation amplifies oxidative stress, perpetuating a harmful cycle that amplifies apoptosis, inflammation, and fibrosis. Caspase 2 and 3 activation, along with increased DNA fragmentation, were markedly upregulated in both patient and animal models of MASLD [130,131]. Consequently, targeted deletion of caspase 2 or 3 has been shown to markedly reduce apoptosis and fibrotic pathways, thereby decelerating disease progression [132,133]. Furthermore, several mediators such as C/EBP homologous protein, protein phosphatase 1 activator, c-Jun N-terminal kinase, and apoptosis signal-regulating kinase 1 have been identified as key contributors to inflammation and fibrosis in the progression of MASLD [1]. A recent study proposed the AMPK-caspase 6 axis as an important target in chronic inflammatory pathogenic processes, including MASH [134]. However, a recent clinical trial reported that the pan-caspase inhibitor emricasan did not improve liver histology in patients with MASH fibrosis and might have contributed to exacerbation of ballooning and fibrosis, suggesting a potential shift from apoptotic cell death to more inflammatory forms such as necroptosis [135]. This example shows the complex balance between different cell death modes and it highlights the necessity to further investigate compensatory cell death mechanisms in preclinical models. 

#### 4.3.3. Necroptosis

In MASLD, necroptosis can be triggered by various factors such as TNF-α [136]. The levels of RIPK3 in the liver increase in patients with MASLD, showing a correlation between hepatic inflammation and fibrosis [137]. Knockdown of RIPK3 in mice led to significant alleviation of obesity, insulin resistance, hepatic steatosis, and oxidative stress, primarily through modulation of lipogenesis-related gene expression and the suppression of the TLR4/NF-κB and NRF2/HO-1 signaling pathways [138]. Inhibiting proteins involved in necroptosis, such as RIPK1 and MLKL, has also shown potential in improving liver steatosis and insulin resistance, critical aspects of MASLD [139]. Moreover, a recent study investigating MASH has defined a canonical role for MLKL as the executor of necroptosis [140]. In a high-fat diet-induced model of MASLD and MASH, liver injury was demonstrated to be dependent on MLKL rather than RIPK3, with MLKL independently attenuating autophagic flux by inhibiting lysosome-autophagosome fusion [140].

#### 4.3.4. Ferroptosis

Iron overload is common among MASLD patients, and it is widely recognized that iron-induced lipid peroxidation serves as both a trigger and a contributor to MASLD [141,142]. Lipid peroxidation products, widely regarded as markers of oxidative stress, including malondialdehyde and 4-hydroxy-2-nonenal (4-HNE), have been significantly elevated in patients with MASLD [143]. Considering the role of ACSL4 in the generation of lipid peroxidases from PUFAs, its expression was notably increased in an animal model of MASH [144]. Further, inhibition of ferroptosis significantly reduced liver inflammation and hepatocyte cell death in early-stage MASH, improving liver function [145]. Enhancing NRF2 expression can prevent MASLD by reducing lipid accumulation and modulating antioxidant responses, highlighting the involvement of ferroptosis in MASLD and the potential of targeting oxidative stress and iron metabolism in treatment strategies. Drugs targeting ferroptosis, such as ferrostatin-1, have been extensively documented, suggesting the potential significance of ferroptosis as a crucial target for MASLD treatment [146]. Despite significant efforts to delineate the characteristics of ferroptosis in MASLD, further research is imperative to clarify its precise and detailed regulatory mechanisms.

#### 4.3.5. Autophagy/Autophagy-Induced Cell Death

Impaired autophagic flux is a hallmark of MASLD [147]. Recent findings elucidate the role of autophagy in regulating hepatocyte lipid metabolism, highlighting its contribution beyond the actions of cytosolic lipases through a process termed macrolipophagy. This mechanism involves the sequestration of lipid droplets within autophagosome vesicles, which are then transported to lysosomes for degradation into fatty acids. The activity of macrolipophagy is closely tied to nutritional status, with an increase in autophagic activity following short-term fat consumption, which can mitigate lipotoxicity. However, prolonged intake of a high-fat diet impedes the fusion of autophagosomes and lysosomes, thereby elevating the risk of MASLD [148]. Indeed, chronic obesity and insulin resistance, as seen in ob/ob mice and high-fat diet models, are associated with the downregulation of autophagy. Impaired autophagy can also contribute to ER stress in lean mice, as the restoration of autophagy ameliorates obesity-induced liver ER stress and improves insulin resistance in vivo, indicating that autophagy facilitates the elimination of damaged organelles and assists in their adaptive response to restore metabolic homeostasis [149].

### 4.4. Hepatocellular Carcinoma

The development of HCC is the final, irreversible step in chronic liver diseases and the most common cause of death in patients with liver cirrhosis [93]. Hepatocarcinogenesis is a multifactorial process wherein cell death plays a fundamental role. The majority of HCCs develop in fibrotic or cirrhotic livers, which arise in the context of chronic hepatocellular death. It is important to differentiate between cell death occurring in benign hepatocytes and cell death occurring in malignant hepatocytes because they may have opposite consequences. Cell death in non-transformed hepatocytes represents a tumor-promoting mechanism, leading to increased compensatory regeneration, fibrogenesis, and inflammation. These compensatory mechanisms contribute to oncogenic transformation, and elevated serum levels of aminotransferases, used as markers for hepatocyte death, are highly predictive of HCC development [150].

#### 4.4.1. Pyroptosis

In general, pyroptosis, inflammasomes, and the gasdermin family contribute to tumorigenesis and cancer progression [151,152,153]. Regarding the liver, several RNA sequencing studies suggest that ‘pyroptosis-related genes’-based risk scores may be useful in predicting the prognosis in HCC patients [154,155]. However, very few studies have investigated the tumor-intrinsic properties of the inflammasome and pyroptosis in HCC. It is described that caspase 1 is downregulated in human HCC tissue; however, no association with prognosis was reported in this study [156]. Similarly, mRNA and protein expression levels of NLRP3, ASC, caspase 1, and IL-1β were decreased in cancerous vs. non-cancerous specimens from healthy and cirrhotic livers [157]. Interestingly, the expression levels were the highest in the cirrhotic, non-cancerous tissue compared to healthy livers and peritumoral hepatitis tissue. In contrast, GSDMD expression was upregulated in fresh frozen samples of tumor vs. non-cancerous tissue from a different patient cohort [158]. In liver cancer cells, the upregulation of pyroptosis-related genes leads to reduced cell migration towards immortalized hepatic stellate cells, indicating that pyroptotic cell death in cancer cells reduces their malignant potential [158]. Pyroptosis in cancer cells might impair their malignant potential, whereas pyroptosis in tumor-adjacent tissue is abundantly present. Cell-specific KOs of inflammasome components are eventually needed to elucidate the underlying signaling pathways of pyroptosis in cancerous and non-cancerous (peritumoral) tissue.

#### 4.4.2. Apoptosis

Under physiological circumstances, hepatocytes rarely turnover, with almost no cell death or proliferation. However, an increased rate of hepatocytes undergoing apoptosis can result in chronic liver injury, eventually leading to tumorigenesis. For instance, mice lacking the anti-apoptotic BCL-2 family member myeloid cell leukemia-1 (MCL-1) in hepatocytes, experience severe liver damage and spontaneously develop liver tumors after several months [159]. Likewise, hepatocyte-specific KO of anti-apoptotic *B-cell lymphoma-extra-large* (BCL-xL), also a member of the BCL-2 family, increases the hepatic TNF-α production, oxidative stress, and tumor incidence. Deletion of pro-apoptotic apoptosis regulator *Bax* reverses these effects and reduces the incidence of liver cancer [160]. Defects in downstream regulators of the apoptotic pathways are critical steps in the malignant transformation of tumor cells, as apoptosis plays a crucial role in safeguarding genomic integrity [4]. For instance, dual deletion of *Ripk1* and *Traf2,* both involved in survival or cell death-promoting pathways, leads to increased apoptosis and compensatory proliferation, promoting spontaneous hepatocarcinogenesis [161]. In patients with HCC undergoing resection or liver transplantation, low expression of RIPK1 and TRAF2 in the tumor was predictive of a poor prognosis [161]. However, considering the intertwined cell death pathways of apoptosis and necroptosis, the notion that RIPK1 solely possesses anti-tumorigenic capabilities is not entirely accurate. In a mouse model with hepatocyte-specific deletion of *transforming growth factor β-activated kinase 1* (TAK1), spontaneous liver fibrosis and tumor development evolved. These processes were abrogated after the deletion of *Ripk1*, indicating a pro-tumorigenic role in this setting [162]. Of note, in this study, apoptosis strongly promoted cell death responses such as inflammation, compensatory hepatocyte proliferation, and carcinogenesis, whereas necroptosis suppressed inflammation, proliferation, and carcinogenesis.

#### 4.4.3. Necroptosis

Necroptosis is associated with necroinflammation in the liver, where persistent inflammation can ignite tumorigenesis. Hepatic necroptosis fosters the recruitment and activation of liver macrophages, inducing chronic inflammation, which subsequently triggers oncogenic pathways, thereby driving the progression of MASLD to HCC in male mice. This effect was abrogated in *Ripk3*^−/−^ or *Mlkl*^−/−^ mice [163]. However, fibrosis was unaffected in this model. New insights into the fine regulation of necroptosis and tumorigenesis were provided by a recently published study. Concurrent activation of the necroptosome and NF-κB activation in hepatocytes, which typically express only low levels of RIPK3 due to their minimal physiological cell turnover, prevented cell death and induced a prolonged ‘sublethal’ state with leaky membranes. Hepatocytes in this sublethal state function as secretory cells releasing specific chemokines, including CCL20 and monocyte chemotactic protein 1 (MCP-1 or CCL2), which promote the activation of pro-carcinogenic monocyte-derived macrophage cell clusters and tumorigenesis. In contrast, inactive NF-κB in the setting of necroptosis accelerated cell death in untransformed hepatocytes and thereby prevented hepatocarcinogenesis [164]. A recent study reported promising results from the treatment of liver cancer cells with CBL0137, a small molecule anti-cancer drug candidate that induces apoptosis and necroptosis [165]. Overall, necroptosis may serve as a tumor-promoting form of cell death that can be regulated within liver cells through NF-κB activation.

#### 4.4.4. Ferroptosis

As described above, ferroptosis is negatively regulated by SLC7A11, GPX4, and GSH. Iron metabolism-related genes such as SLC7A11 are upregulated in human cancers and are associated with a poor prognosis in HCC [166,167]. Ferroptosis may serve as a predominant driver of HCC-promoting necroinflammation in MASH-HCC. Ectopic SLC7A11 expression suppressed HCC development in hepatocyte-specific *Atf4*-deficient mice and provided protection from ferroptosis. This suggests that ferroptosis contributes to the necroinflammatory response that promotes HCC and indicates that ferroptosis inhibitors may prevent the progression from MASH to HCC [168]. In a human HCC cell line (HepG2), sorafenib-mediated cell death was blocked by ferrostatin-1 but not ZVAD-FMK (an apoptosis inhibitor) and necrosulfonamide (a necroptosis inhibitor). Notably, treatment with sorafenib and other ferroptosis-inducing agents upregulated NRF2, which promotes SLC7A11 transcription. In vivo experiments with NRF2 knockdown tumor cells showed reduced tumor size, an effect further enhanced with additional sorafenib treatment [169]. SLC7A11, a crucial component of the cystine/glutamate antiporter that blocks ferroptosis, is overexpressed in various human cancers, including liver cancer [170]. Interestingly, the tumor suppressor p53 downregulates SLC7A11 expression, rendering cancer cells more susceptible to ferroptotic cell death [170]. The expression of GPX4, another ferroptotic inhibitor, was observed in human hepatocellular carcinoma specimens and correlated with a poor survival prognosis [171].

#### 4.4.5. Autophagy/Autophagy-Induced Cell Death

Autophagy is commonly recognized for its dual impact on both tumorigenesis and cancer advancement. Initially, autophagy functions as a tumor suppressor by impeding tumorigenesis through the elimination of damaged cell organelles and the mitigation of oxidative stress, thereby reducing DNA damage and genome instability. However, once normal cells transform into tumor cells, autophagy can be upregulated to survive microenvironmental stress and increase tumor growth [172]. In human liver tumors, p62, a ubiquitin-binding autophagy receptor that accumulates when autophagy is impaired, is highly expressed [173]. Furthermore, elevated expression of p62 in hepatocytes enhances HCC induction, and its high expression in non-tumor human liver predicts rapid HCC recurrence after curative ablation [174]. LAMP2A, a regulator of chaperon-mediated autophagy, exhibits reduced expression in human tumor tissues compared to adjacent non-tumor tissues. Additionally, LAMP2A downregulation significantly increased cell proliferation in human hepatocyte and HCC cell lines [175]. Phosphatase and tensin homolog (PTEN) deletion in the liver activates the AKT/mTOR pathway, which induces autophagy inhibition and increases protein synthesis, both of which enhance ER stress [176]. Apart from its role in hepatocytes, autophagy is involved in the activation of HSCs, a hallmark of fibrogenesis in the liver [177]. The suppression of autophagy in HSCs attenuates the activation of HSCs in vivo and leads to the suppression of fibrosis and tumor formation [178]. While p62 has a tumor-promoting effect in hepatocytes, whole-body depletion, and HSCs-specific depletion leads to enhanced tumorigenesis, implying an overall tumor-suppressive dominant role in HSCs [179].

### 4.5. Tumor Microenvironment

In the previous section, we explored the existing evidence regarding the role of regulated cell death in hepatic tumor development and progression. However, tumorigenesis regularly occurs in conjunction with alterations in the surrounding stroma, creating a heterogeneous landscape known as the tumor microenvironment (TME). In the liver, TME plays a crucial role, as most HCCs arise from chronically injured hepatocytes in cirrhotic livers. Consequently, the TME has emerged as an essential driver contributing to tumor survival, growth, angiogenesis or cell invasion, and the maintenance of hepatocarcinogenesis [180]. The TME is orchestrated by both cellular and non-cellular components, including cancer cells, stromal tissue, and the surrounding matrix. Stromal components include immune cells, fibroblasts, angiogenic cells, and vascular tissue, which collectively play an active role in eliciting inflammatory responses. The focal point of interest lies in active communication between stromal cells (recruited or resident), cancer cells, and proximal immune cells. This interaction occurs either directly or indirectly through the production of inhibitory/stimulatory signals, underscoring the significant role of the TME in the pathogenesis of HCC. Regulated cell death processes play a pivotal role in intercellular communication by the release of DAMPs and cytokines.

#### 4.5.1. Pyroptosis

It has been demonstrated that cancer cells undergoing epithelial-mesenchymal transition (EMT), a hallmark of cancer, suppress NLRP3 inflammasome activities of tumor-associated macrophages (TAMs) in response to chemotherapy [181]. This suppression is mediated through the delivery of exosomal miR-21. Consequently, the inactivation of the NLRP3 inflammasome lowers IL-1β secretion, thereby reducing chemotherapy-induced inflammation and cell death in the TME of patients with head and neck cancer [181]. In breast cancer, cancer-associated fibroblasts (CAF), another abundant cell type in the TME, facilitate tumor growth and metastasis through inflammasome signaling, a process attenuated when NLRP3 or IL-1β is specifically ablated [182]. In liver cancer, transcriptomic data from HCC samples indicate a highly immunosuppressive TME and upregulated markers for apoptosis, ferroptosis, and pyroptosis are associated with poorer survival [183]. However, it remains unclear how pyroptosis precisely affects biological processes in the TME of HCC and contributes to a pro- or anti-immunogenic TME.

#### 4.5.2. Apoptosis

Understanding how apoptosis is regulated in different cell types in the TME presents a significant challenge. Typically, apoptotic pathways are downregulated in malignant hepatocytes, another typical feature of cancer cells. Therefore, inducing apoptosis in cancer cells is a therapeutic strategy. However, apoptosis and cell death induction in immune cells located within the TME are associated with chemotherapy resistance and immune evasion by HCC cells. Given that HCC commonly arises in pre-damaged, fibrotic livers, there may be benefits to inducing apoptosis and clearing activated HSCs, which are also present in the TME. For instance, the interaction between HCC and HSCs has been shown to promote sorafenib resistance in HCC cells. Hence, pharmacological inhibition of HSC activation increases apoptotic cell death in sorafenib-treated multicellular hepatic spheroid containing HSCs and HCC cells [184]. In patients with HCC, higher expression of marker genes of apoptosis, ferroptosis, and pyroptosis have been linked to an immunosuppressive TME and poorer survival outcomes [183].

#### 4.5.3. Necroptosis

Single-cell RNA sequencing analysis shows that tumor-associated macrophages are prone to undergo necroptosis. In addition, spatial transcriptomics data reveals their co-localization with poorly differentiated tumor regions in HCC. Interestingly, in the absence of a necroptotic microenvironment, pre-malignant cells could evolve into higher malignant HCC subtypes [185]. Another study found that a necroptosis-associated hepatic cytokine microenvironment triggers intrahepatic cholangiocarcinoma (ICC) development independently of the oncogenic drivers [186]. The authors used hydrodynamic tail vein injection (HDTV)-mediated transposon delivery and electroporation (Epo) to induce liver tumors. While HDTV induced HCC, electroporation, which induces surrounding liver damage and necrosis, led to ICC. Treatment with necrostatin-1, a potent necroptosis inhibitor, shifted cell death to apoptosis, attenuated Epo-associated cytokine induction, and redirected ICC development towards the outgrowth of solid HCC tumors. The results were confirmed using mice deficient for *Mlkl* in hepatocytes, indicating that a necroptosis-enriched liver microenvironment promotes ICC outgrowth from oncogenically transformed hepatocytes.

#### 4.5.4. Ferroptosis

New insights into the role of ferroptosis in tumorigenesis and TME were provided by a study conducted by Conche et al. [187]. GPX4, a master regulator of ferroptosis, was knocked out in hepatocytes, inducing ferroptosis in vitro as well as in vivo, resulting in acute liver failure and inflammation. Meanwhile, the induction of ferroptosis in HCC was insufficient to suppress tumorigenesis. Ferroptosis in non-malignant hepatocytes induces an anti-tumor response through CXCL10 secretion, stimulating cytotoxic CD8^+^ T cells into the tumor. This anti-tumor response from ferroptotic hepatocytes was counterbalanced by the simultaneous release of HMGB1, a cellular DAMP, from tumor cells. HMGB1 triggered the infiltration of myeloid-derived suppressor cells (MDSC) in conjunction with programmed death-ligand 1 (PD-L1) upregulation, leading to immunosuppression and protecting tumor cells from the ferroptosis-induced CD8-dependent anti-tumor activity. Consequently, the combined blockade of PD-1 and MDSC infiltration in HCC conferred a survival benefit in mice. Interestingly, primary colorectal cancer did not respond to this approach, suggesting that the ferroptosis-induced immune response is specific for the respective local TME. Besides contributing to our understanding of ferroptosis in HCC, the study unravels the complexity of cell death in the context of tumor development, tumor progression, and TME. Cell death in both benign and malignant liver cells can have pro- or anti-tumorigenic effects or both simultaneously. It seems likely that dying or sublethal cells are simultaneously secreting a myriad of DAMPs and cytokines, balancing the reaction of the extracellular microenvironment beyond a simple ‘pro-’ or ‘anti-tumor’ effect.

#### 4.5.5. Autophagy/Autophagy-Induced Cell Death

The growth, metastasis, and recurrence of hepatocellular carcinoma are driven and sustained by liver cancer stem cells (LCSCs), potentially contributing to the unfavorable prognosis associated with the disease. These cells thrive in a TME marked by common features such as oxygen and nutrient deficiencies. It is likely that autophagy plays a role in maintaining CD133^+^ LCSCs under conditions of oxygen and nutrient deprivation [188]. The autophagy inhibitor chloroquine has been shown to increase apoptosis and reduce cell division of LCSCs, potentially sensitizing them to the TME and improving the efficacy of anti-cancer treatments. In a study involving patients undergoing curative resection for HCC, high expression of LC3, an autophagy-related marker, in both the tumor and the TME was significantly associated with lower recurrence rates, suggesting a tumor-suppressive function of autophagy in both tumor tissue and the TME [189].

### 4.6. Liver Surgery/Ischemia-Reperfusion Injury

Hepatic ischemia-reperfusion injury (IRI) is a major complication of liver resection and liver transplantation. IRI occurs when the blood supply to the liver is interrupted, leading to hypoxia and cell damage. In liver resection, IRI occurs after hepatic portal occlusion (HPO or Pringle maneuver), while in liver transplantation, warm and cold ischemia times contribute to IRI, affecting the organ quality and amplifying inflammatory processes [190]. Paradoxically, the restoration of the blood flow, the reperfusion, exacerbates tissue damage due to the preceding oxygen deprivation [191]. Oxidative stress leads to the activation of inflammatory pathways and mitochondrial damage, culminating in excessive cell death of parenchymal liver cells [192]. Therefore, IRI can affect all stages of liver transplantation and impact the postoperative outcome after liver surgery.

#### 4.6.1. Pyroptosis

Pyroptosis has been shown to play a role in various transplanted organs, including kidneys and the heart [193,194]. In a porcine liver transplantation model, the comparison of cold storage vs. different protocols of machine perfusion strongly suggests a role for NLRP3- and IL-18-related pathways [195,196,197]. Inhibiting NLRP3 by the small-molecule inhibitor MCC950, either added to the hypothermic machine perfusion system or injected after transplantation, reduced pyroptosis, and improved outcomes in pigs [198]. In a murine IRI study of warm ischemia, the expression levels of nucleotide-binding oligomerization domain 1 (NOD1), caspase 1, and GSDMD increased significantly [199]. Treatment with a NOD1 agonist before induction of IRI led to an increased expression of pyroptosis-related genes. Pre-damaged steatotic livers are even more prone to IRI damage and express higher levels of cleaved caspase 1 and GSDMD [200]. Newer evidence demonstrated the potential to target and address cell death regulators in the context of IRI. In cells undergoing pyroptosis or apoptosis, cell-surface protein NINJ1 is crucial for plasma membrane rupture. Deficiency or inhibition of NINJ1 improved hepatocellular plasma membrane integrity in an IRI model, resulting in reduced liver enzymes, decreased IL-18 and DAMPs such as HMGB1, and diminished neutrophil infiltration [6]. Cell-specific findings showed that NINJ1 deficiency in Kupffer cells protected mice from IRI through NINJ1/DUSP1 signaling, reducing neutrophil infiltration. These results were validated in a small human patient cohort undergoing liver resection with portal occlusion, where preoperative sivelestat, a neutrophil elastase inhibitor, decreased postoperative serum levels of myeloperoxidase and liver enzymes, highlighting the critical role of enhanced neutrophil activity in hepatic IRI, influenced by cell death in resident macrophages [201].

#### 4.6.2. Apoptosis

Most of the described mechanisms of IRI generally assume cell damage that primarily leads to lytic cell death. However, several reports propose that apoptosis occurs in the human and rodent post-ischemic liver [202,203,204]. Apoptosis during IRI is initiated through the activation of death receptors, specifically through the signaling pathways involving the death receptor CD95 (Apo-1/Fas) and its corresponding ligand CD95L [205]. The neutralization of CD95L not only protects the liver against IRI but also attenuates liver damage, reducing both apoptosis and necrosis [206]. In patients who have undergone liver resection with HPO compared to those without, an increased rate of apoptosis is observed [207].

#### 4.6.3. Necroptosis

Overall evidence suggests that necroptosis plays a relevant role as a form of lytic cell death in the IRI model, although some studies do not attribute any effects to necroptosis [208,209]. A recent study revealed the presence of activated MLKL in human liver grafts before and after reperfusion [210]. Interestingly, patients experiencing early allograft dysfunction show a significant increase in MLKL activation, which correlates with serum liver enzyme levels. Additionally, IRI damage is increased in pre-damaged steatotic livers, as liver steatosis exacerbates hepatic IR injury through increased MLKL-mediated necroptosis both in in vivo and in vitro models [211,212]. Furthermore, it exacerbates liver damage after IRI in aging mice [213]. Mechanistically, MLKL deficiency may promote PTEN-induced kinase 1 (PINK1)-mediated mitophagy activation, inhibiting oxidative DNA damage in hepatocytes, which in turn suppresses macrophage cGAS-STING activation and inflammatory liver IRI [214]. Underlining the importance of necroptosis as a mediator of intercellular crosstalk, it is suggested that MLKL-mediated hepatocyte necroptosis is controlled by the Notch pathway in macrophages [215]. Despite conflicting evidence, overall data suggest a detrimental role of necroptosis in IRI.

#### 4.6.4. Ferroptosis

In a recent study using a murine IRI model, it was discovered that transmembrane member 16A (TMEM16A), a component of hepatocyte Ca^2+^-activated chloride channels, interacts with GPX4, leading to its ubiquitination and degradation, thereby promoting ferroptosis [216]. Knocking out TMEM16A, specifically in hepatocytes in mice, reduced IRI-induced liver damage, improving inflammation and ferroptotic cell death. Analysis of liver specimens from patients undergoing liver resection with hepatic portal occlusion revealed increased TMEM16A post-resection compared to pre-resection biopsies. In a different patient cohort, intraoperative hepatic portal occlusion reduced GPX4 expression and increased iron overload in the liver [207]. Consistent with these findings, mice fed a high-iron diet exhibited exacerbated IRI, suggesting that targeting ferroptosis could be a viable strategy to mitigate IRI in both mice and patients.

#### 4.6.5. Autophagy/Autophagy-Induced Cell Death

Autophagy plays a crucial role in cellular adaptation to various endogenous and exogenous stressors, including IRI [217]. In a murine IRI model, increased levels of LC3-II and p62 proteins suggest either autophagosome formation or impaired fusion with lysosomes, leading to their accumulation [218]. Mild hypothermia treatment, used to mitigate organ damage in transplantation, upregulates Rab7 expression facilitating autophagosome degradation and reducing liver damage. Mitophagy, the selective removal of damaged mitochondria, is protective in hepatocytes, although its mechanisms remain incompletely understood. In rats, the anti-apoptotic augmenter of liver regeneration (ALR) protein, found in mitochondria, promotes mitophagy by recruiting PINK1 and Parkin to mitochondria, thereby attenuating IRI [219]. Overall, autophagy in injured liver cells might protect from cell death in the context of IRI. However, more studies are needed to fully understand the mechanisms.

## 5. Potential Treatment Options: Targeting Cell Death in Liver Diseases

Over the past few decades, numerous researchers have investigated cell death pathways using inhibitors and methodologies in both animal models and human studies to identify clinically applicable drugs that can mitigate liver diseases (Table 1). In this chapter, we provide a summary of treatment options based on their respective targets for cell death, along with an overview of potential applications in various liver diseases.

### 5.1. Pyroptosis

In recent years, a number of small molecular compounds, such as CY-09, MCC950, oridonin, IFM-514, and tranilast, have demonstrated efficacy in mitigating inflammatory diseases through the inhibition of the NLRP3 inflammasome [247]. CY-09 reduces hepatic steatosis and insulin resistance, whether used alone or in combination with endoscopic sleeve gastroplasty, in a MASLD animal model [248,258]. MCC950 improves steatosis and fibrosis in elderly mice as well as in a MASLD animal model, accompanied by normalized hepatic caspase 1 and IL-1β expression [249,250]. The NLRP3 blocker oridonin has also been reported to attenuate inflammatory cell infiltration and injury in the ALD model and ameliorate CCL_4_-induced liver fibrosis [251,252]. Disulfiram, clinically used to treat alcohol use disorder, has recently been demonstrated to inhibit pyroptosis by blocking GSDMD pore formation [259]. Treatment with disulfiram alleviates acute lung injury and ischemia/reperfusion-induced acute kidney injury, indicating a potential application in liver disease beyond alcohol use disorder [260,261]. The therapeutic benefits of the IL-1β antibody canakinumab for the treatment of alcoholic hepatitis are currently investigated. The randomized-controlled trial NCT03775109 is testing wether canakinumab can improve histology and survival rates [253].

### 5.2. Apoptosis

Previously, the pan-caspase inhibitor VX-166 showed conflicting results in the reduction of hepatic steatosis, histological inflammation, and liver injury across different dietary models [220,221]. The pan-caspase inhibitor emricasan has been reported to mitigate liver injury and fibrosis through the inhibition of apoptosis in a MASLD animal model [222]. However, its beneficial effect in preclinical models was not confirmed in clinical trials including patients with MASH and MASH-related cirrhosis [135,225]. Treatment with GS-9450 clinically induced reductions in liver enzyme levels, but it did not significantly decrease cytokeratin 18, which is released from apoptotic cells, in sera from patients with MASH [224]. On the other hand, three months of administration of aramchol, which regulates SCD1 fatty acid enzymes, has been reported to be safe, tolerable, and significantly reduce liver fat content in patients with MASLD, potentially targeting ER-stress and apoptosis [226].

### 5.3. Necroptosis

Necrostatin-1, a potent RIPK1 inhibitor, has protective effects against hepatic injury, improving liver function and reducing inflammatory responses in animal models of liver IRI and drug-induced liver failure [243,244]. Interestingly, necrostatin-1 also exhibits an off-target effect by suppressing ferroptosis at high concentrations, although the mechanism behind it is not yet fully understood [262]. The RIPK1 inhibitor R552, which has completed phase I clinical trials and is currently undergoing phase II studies for autoimmune and inflammatory diseases, has clear effects on liver disease [263]. Another promising inhibitor, RIPA-56, a highly specific and metabolically stable inhibitor of RIPK1, improved liver inflammation and fibrosis in mice fed a high-fat diet and was effective in hepatocytes from patients with MASLD [245]. Additionally, the novel MLKL inhibitor, P28, demonstrated potent anti-fibrotic properties by reducing the activation of hepatic stellate cells and expression of fibrosis-related markers without having cytotoxic effects [264].

### 5.4. Ferroptosis

Targeting ferroptosis might be a promising therapeutic option in various disorders, especially liver diseases. Prior reports suggest that iron chelators like deferoxamine and ciclopirox olamine can curb lipid peroxidation propagation by preventing the Fenton reaction, although research on their efficacy in liver diseases is limited [20]. Drugs that target lipid peroxidation, such as ferrostatin-1, liproxstatin-1, and vitamin E, show promise in alleviating liver dysfunction and ferroptosis in different animal models of liver diseases, including acute liver injury, autoimmune hepatitis, ALD, and MASLD [115,254,256,265]. Interestingly, liproxstatin-1, a ferroptosis inhibitor, has been found to alleviate steatosis and steatohepatitis in MASLD by broadly mitigating cell death pathways involving apoptosis, pyroptosis, and necroptosis, hinting at a potential link between ferroptosis and other forms of cell death [255]. Additionally, targeting ACSL4 with thiazolidinediones, a class of antidiabetic compounds, has shown promise in inhibiting ferroptosis in a lipoxygenase-dependent manner, aligning with their recommended use in MASLD [23]. FGF21, a biological analog, has recently emerged as a novel ferroptosis suppressor, expanding the treatment options for diseases associated with ferroptosis [257]. Despite the growing research on ferroptosis modulators in various diseases, further investigations are needed to fully elucidate the roles and mechanisms of these pharmacological agents in liver diseases.

### 5.5. Autophagy/Autophagy-Induced Cell Death

Pharmacological strategies targeting autophagy have been explored for liver disease treatment. Suppression of mTORC1 by rapamycin or everolimus increases peroxisome proliferator-activated receptor α (PPARα) activity and autophagy, reducing steatosis and inflammation in MASLD models [231,232]. However, these interventions affect various pathways, and their impact on fibrosis remains controversial. The AMPK activator metformin has been reported to improve ALD and MASLD through the upregulation of mitophagy [234,235]. Treatment with ezetimibe, which is usually used to treat hypercholesterolemia, improves steatohepatitis by promoting autophagy via AMPK activation and TFEB nuclear translocation, independent of mTOR [236]. FGF21 analogs improve insulin sensitivity and reduce liver fat in MASH patients [266]. Stimulating SIRT3 with agents like dihydromyricetin or berberine, along with activating the NRF2/FXR/LXRα pathway with curcumin, benefits MASLD-related conditions [238,239,240]. The novel NRF2 activator S217879 rectifies autophagy defects and enhances liver health in MASLD progression [242]. Despite ongoing efforts, no autophagy-selective drugs are in clinical development, highlighting the need for further research to find potent and safe autophagy modulators for liver diseases.

## 6. Conclusions

We have reviewed and summarized various forms of programmed cell death and related molecular mechanisms in the context of liver diseases. Different types of programmed cell death play a unique role in maintaining liver function. However, disruption in any of these mechanisms can contribute to the onset of liver diseases. Our comprehension of cell death pathways as initiators and modulators of disease is continually growing. Envisioning the targeted regulation of key players in cell death pathways for disease treatment and cellular damage alleviation is intriguing. However, evidence also highlights the need to consider the intricate and complex network of intertwined and compensatory pathways. For instance, in liver cancer, the response of both tumoral and non-tumoral microenvironments to dying cells must be taken into account. Many cell death pathways are counterbalanced by the activation of complementary pathways, and the release of various DAMPs can elicit diverse reactions in distinct cell types. Understanding how cell death pathways operate in various cell types under different pathological situations remains to be elucidated. For precise treatment of liver diseases, a combination of distinct inhibitors may be necessary to address the multitude of pathways, ultimately enabling the provision of precise and effective treatment options for patients. Despite significant advances in liver disease research, several challenges must be addressed before the regulation of cell death can be effectively utilized as a therapeutic strategy in clinical settings. Given the diverse range of pathogenic factors that trigger cell death programs and the complexity of liver disease pathogenesis, a detailed understanding of the interactions between different forms of programmed cell death and their impact on disease progression is imperative. This approach could ultimately facilitate the development of accurate and effective treatment options for patients.

## Figures and Tables

**Table 1:** Pharmacologic Inhibitors of Cell Death in Liver Disease.

Cell Death	Drug	Stage	Target of Compound	Disease/Model	Ref.
**Apoptosis**	VX-166	Preclinical	Pan-caspase	MASLD/MASH Cirrhosis	[135,220,221,222,223,224,225]
	GS-9450	Phase II, randomized, placebo-controlled trial			
	Emricasan (IDN-6556)	Randomized, placebo-controlled trial			
	Aramchol	Randomized, placebo-controlled trial	Stearoyl-CoA desaturase 1	MASLD	[226]
	Meretrix oligopeptides	Preclinical	NF-κB	MASLD	[227]
	Seladelpar (MBX-8025)	Preclinical	Proliferator-activated receptor-delta	MASH	[228]
	Resveratrol	Preclinical	SIRT1	MASLD	[229]
	TBE-31	Preclinical	NRF2	MASH/Fibrosis	[230]
**Autophagy**	Rapamycin	Preclinical	mTORC1	MASLD/MASH Liver transplantation with HCC	[231,232,233]
	Everolimus	Randomized trial			
	Metformin	Preclinical	AMPK	ALD MASLD	[234,235]
	Ezetimibe	Preclinical	AMPK, TFEB	MASH	[236]
	FGF21	Randomized, placebo-controlled trial	AMPK	MASLD/MASH	[237]
	Dihydromyricetin Berberine	Preclinical	SIRT3	MASLD	[238,239]
	Curcumin	Preclinical	NRF2/FXR/LXRα	MASLD ALD	[240,241]
	S217879	Preclinical	NRF2	MASLD	[242]
**Necroptosis**	Necrostatin-1	Preclinical	RIPK1	Liver I/R injury Drug-induced acute liver injury	[243,244]
	RIPA-56	Preclinical	RIPK1	MASLD/MASH	[245]
	Necrosulfonamide	Preclinical	MLKL	CCL_4_-induced acute injury	[246]
**Pyroptosis**	CY-09, Tranilast, Oridonin MCC950 IFM-514	Preclinical	NLRP3	MASLD/MASH ALD Liver fibrosis	[247,248,249,250,251,252]
	Canakinumab	Randomized, placebo-controlled trial	IL-1β	Alcoholic hepatitis	[253]
**Ferroptosis**	Ferrostain-1, Liproxstatin-1 Vitamin E	Preclinical	Lipophilic antioxidation	TAA-induced ALI Autoimmune hepatitis MASLD/MASH ALD	[115,254,255,256]
	Selenium	Preclinical	GPX4	MASH	[145]
	FGF21	Preclinical	HO-1 inhibition, NRF2 activation	Iron overload-induced liver damage and fibrosis	[257]

## References

[1] Schwabe R.F., Luedde T. (2018). Apoptosis and necroptosis in the liver: A matter of life and death. Nat. Rev. Gastroenterol. Hepatol..

[2] Stoess C., Leszczynska A., Kui L., Feldstein A.E. (2023). Pyroptosis and gasdermins—Emerging insights and therapeutic opportunities in metabolic dysfunction-associated steatohepatitis. Front. Cell Dev. Biol..

[3] Galluzzi L., Vitale I., Aaronson S.A., Abrams J.M., Adam D., Agostinis P., Alnemri E.S., Altucci L., Amelio I., Andrews D.W. (2018). Molecular mechanisms of cell death: Recommendations of the Nomenclature Committee on Cell Death 2018. Cell Death Differ..

[4] Vitale I., Pietrocola F., Guilbaud E., Aaronson S.A., Abrams J.M., Adam D., Agostini M., Agostinis P., Alnemri E.S., Altucci L. (2023). Apoptotic cell death in disease—Current understanding of the NCCD 2023. Cell Death Differ..

[5] Knorr J., Wree A., Feldstein A.E. (2022). Pyroptosis in Steatohepatitis and Liver Diseases. J. Mol. Biol..

[6] Kayagaki N., Stowe I.B., Alegre K., Deshpande I., Wu S., Lin Z., Kornfeld O.S., Lee B.L., Zhang J., Liu J. (2023). Inhibiting membrane rupture with NINJ1 antibodies limits tissue injury. Nature.

[7] Kayagaki N., Kornfeld O.S., Lee B.L., Stowe I.B., O’Rourke K., Li Q., Sandoval W., Yan D., Kang J., Xu M. (2021). NINJ1 mediates plasma membrane rupture during lytic cell death. Nature.

[8] Lawlor K.E., Khan N., Mildenhall A., Gerlic M., Croker B.A., D’Cruz A.A., Hall C., Kaur Spall S., Anderton H., Masters S.L. (2015). RIPK3 promotes cell death and NLRP3 inflammasome activation in the absence of MLKL. Nat. Commun..

[9] Kang T.B., Yang S.H., Toth B., Kovalenko A., Wallach D. (2013). Caspase-8 blocks kinase RIPK3-mediated activation of the NLRP3 inflammasome. Immunity.

[10] Shojaie L., Iorga A., Dara L. (2020). Cell Death in Liver Diseases: A Review. Int. J. Mol. Sci..

[11] Luedde T., Kaplowitz N., Schwabe R.F. (2014). Cell death and cell death responses in liver disease: Mechanisms and clinical relevance. Gastroenterology.

[12] Vercammen D., Beyaert R., Denecker G., Goossens V., Van Loo G., Declercq W., Grooten J., Fiers W., Vandenabeele P. (1998). Inhibition of caspases increases the sensitivity of L929 cells to necrosis mediated by tumor necrosis factor. J. Exp. Med..

[13] Oberst A., Dillon C.P., Weinlich R., McCormick L.L., Fitzgerald P., Pop C., Hakem R., Salvesen G.S., Green D.R. (2011). Catalytic activity of the caspase-8-FLIP(L) complex inhibits RIPK3-dependent necrosis. Nature.

[14] Lin Y., Devin A., Rodriguez Y., Liu Z.G. (1999). Cleavage of the death domain kinase RIP by caspase-8 prompts TNF-induced apoptosis. Genes Dev..

[15] Tummers B., Green D.R. (2017). Caspase-8: Regulating life and death. Immunol. Rev..

[16] Moriwaki K., Chan F.K. (2016). Regulation of RIPK3- and RHIM-dependent Necroptosis by the Proteasome. J. Biol. Chem..

[17] Galluzzi L., Kepp O., Chan F.K., Kroemer G. (2017). Necroptosis: Mechanisms and Relevance to Disease. Annu. Rev. Pathol..

[18] Quarato G., Guy C.S., Grace C.R., Llambi F., Nourse A., Rodriguez D.A., Wakefield R., Frase S., Moldoveanu T., Green D.R. (2016). Sequential Engagement of Distinct MLKL Phosphatidylinositol-Binding Sites Executes Necroptosis. Mol. Cell.

[19] Taniguchi T., Matsuo N., Ishikura H., Moriguchi T., Yamamoto T., Tanaka T. (1989). [Platelet volumetry and functional significance in septic rat: Alterations in adenylate pool: Preliminary report]. Nihon Geka Gakkai Zasshi.

[20] Dixon S.J., Lemberg K.M., Lamprecht M.R., Skouta R., Zaitsev E.M., Gleason C.E., Patel D.N., Bauer A.J., Cantley A.M., Yang W.S. (2012). Ferroptosis: An iron-dependent form of nonapoptotic cell death. Cell.

[21] Stockwell B.R. (2022). Ferroptosis turns 10: Emerging mechanisms, physiological functions, and therapeutic applications. Cell.

[22] Dixon S.J., Winter G.E., Musavi L.S., Lee E.D., Snijder B., Rebsamen M., Superti-Furga G., Stockwell B.R. (2015). Human Haploid Cell Genetics Reveals Roles for Lipid Metabolism Genes in Nonapoptotic Cell Death. ACS Chem. Biol..

[23] Doll S., Proneth B., Tyurina Y.Y., Panzilius E., Kobayashi S., Ingold I., Irmler M., Beckers J., Aichler M., Walch A. (2017). ACSL4 dictates ferroptosis sensitivity by shaping cellular lipid composition. Nat. Chem. Biol..

[24] Yang W.S., SriRamaratnam R., Welsch M.E., Shimada K., Skouta R., Viswanathan V.S., Cheah J.H., Clemons P.A., Shamji A.F., Clish C.B. (2014). Regulation of ferroptotic cancer cell death by GPX4. Cell.

[25] Doll S., Freitas F.P., Shah R., Aldrovandi M., da Silva M.C., Ingold I., Goya Grocin A., Xavier da Silva T.N., Panzilius E., Scheel C.H. (2019). FSP1 is a glutathione-independent ferroptosis suppressor. Nature.

[26] Mao C., Liu X., Zhang Y., Lei G., Yan Y., Lee H., Koppula P., Wu S., Zhuang L., Fang B. (2021). DHODH-mediated ferroptosis defence is a targetable vulnerability in cancer. Nature.

[27] Kraft V.A.N., Bezjian C.T., Pfeiffer S., Ringelstetter L., Muller C., Zandkarimi F., Merl-Pham J., Bao X., Anastasov N., Kossl J. (2020). GTP Cyclohydrolase 1/Tetrahydrobiopterin Counteract Ferroptosis through Lipid Remodeling. ACS Cent. Sci..

[28] Park E., Chung S.W. (2019). ROS-mediated autophagy increases intracellular iron levels and ferroptosis by ferritin and transferrin receptor regulation. Cell Death Dis..

[29] Li X., Chen J., Yuan S., Zhuang X., Qiao T. (2022). Activation of the P62-Keap1-NRF2 Pathway Protects against Ferroptosis in Radiation-Induced Lung Injury. Oxidative Med. Cell. Longev..

[30] Wang Y., Tang B., Zhu J., Yu J., Hui J., Xia S., Ji J. (2022). Emerging Mechanisms and Targeted Therapy of Ferroptosis in Neurological Diseases and Neuro-oncology. Int. J. Biol. Sci..

[31] Cheng X.W., Li J., Zhang L., Hu W.J., Zong L., Xu X., Qiao J.P., Zheng M.J., Jiang X.W., Liang Z.K. (2021). Identification of SARS-CoV-2 Variants and Their Clinical Significance in Hefei, China. Front. Med..

[32] Dang Q., Sun Z., Wang Y., Wang L., Liu Z., Han X. (2022). Ferroptosis: A double-edged sword mediating immune tolerance of cancer. Cell Death Dis..

[33] Poznanski S.M., Singh K., Ritchie T.M., Aguiar J.A., Fan I.Y., Portillo A.L., Rojas E.A., Vahedi F., El-Sayes A., Xing S. (2021). Metabolic flexibility determines human NK cell functional fate in the tumor microenvironment. Cell Metab..

[34] Liu Y., Duan C., Dai R., Zeng Y. (2021). Ferroptosis-mediated Crosstalk in the Tumor Microenvironment Implicated in Cancer Progression and Therapy. Front. Cell Dev. Biol..

[35] Qian H., Chao X., Williams J., Fulte S., Li T., Yang L., Ding W.X. (2021). Autophagy in liver diseases: A review. Mol. Asp. Med..

[36] Zachari M., Ganley I.G. (2017). The mammalian ULK1 complex and autophagy initiation. Essays Biochem..

[37] Takahashi Y., He H., Tang Z., Hattori T., Liu Y., Young M.M., Serfass J.M., Chen L., Gebru M., Chen C. (2018). An autophagy assay reveals the ESCRT-III component CHMP2A as a regulator of phagophore closure. Nat. Commun..

[38] Eskelinen E.L. (2006). Roles of LAMP-1 and LAMP-2 in lysosome biogenesis and autophagy. Mol. Asp. Med..

[39] Chao X., Qian H., Wang S., Fulte S., Ding W.X. (2020). Autophagy and liver cancer. Clin. Mol. Hepatol..

[40] Boada-Romero E., Martinez J., Heckmann B.L., Green D.R. (2020). The clearance of dead cells by efferocytosis. Nat. Rev. Mol. Cell Biol..

[41] Lauber K., Blumenthal S.G., Waibel M., Wesselborg S. (2004). Clearance of apoptotic cells: Getting rid of the corpses. Mol. Cell.

[42] Idzko M., Ferrari D., Eltzschig H.K. (2014). Nucleotide signalling during inflammation. Nature.

[43] Lauber K., Bohn E., Krober S.M., Xiao Y.J., Blumenthal S.G., Lindemann R.K., Marini P., Wiedig C., Zobywalski A., Baksh S. (2003). Apoptotic cells induce migration of phagocytes via caspase-3-mediated release of a lipid attraction signal. Cell.

[44] Gude D.R., Alvarez S.E., Paugh S.W., Mitra P., Yu J., Griffiths R., Barbour S.E., Milstien S., Spiegel S. (2008). Apoptosis induces expression of sphingosine kinase 1 to release sphingosine-1-phosphate as a “come-and-get-me” signal. FASEB J..

[45] Chen G.Y., Nunez G. (2010). Sterile inflammation: Sensing and reacting to damage. Nat. Rev. Immunol..

[46] Wang J., Kubes P. (2016). A Reservoir of Mature Cavity Macrophages that Can Rapidly Invade Visceral Organs to Affect Tissue Repair. Cell.

[47] Elliott M.R., Chekeni F.B., Trampont P.C., Lazarowski E.R., Kadl A., Walk S.F., Park D., Woodson R.I., Ostankovich M., Sharma P. (2009). Nucleotides released by apoptotic cells act as a find-me signal to promote phagocytic clearance. Nature.

[48] Shi J., Gao W., Shao F. (2017). Pyroptosis: Gasdermin-Mediated Programmed Necrotic Cell Death. Trends Biochem. Sci..

[49] McDonald B., Pittman K., Menezes G.B., Hirota S.A., Slaba I., Waterhouse C.C., Beck P.L., Muruve D.A., Kubes P. (2010). Intravascular danger signals guide neutrophils to sites of sterile inflammation. Science.

[50] Marques P.E., Amaral S.S., Pires D.A., Nogueira L.L., Soriani F.M., Lima B.H., Lopes G.A., Russo R.C., Avila T.V., Melgaco J.G. (2012). Chemokines and mitochondrial products activate neutrophils to amplify organ injury during mouse acute liver failure. Hepatology.

[51] Antoniades C.G., Quaglia A., Taams L.S., Mitry R.R., Hussain M., Abeles R., Possamai L.A., Bruce M., McPhail M., Starling C. (2012). Source and characterization of hepatic macrophages in acetaminophen-induced acute liver failure in humans. Hepatology.

[52] Dal-Secco D., Wang J., Zeng Z., Kolaczkowska E., Wong C.H., Petri B., Ransohoff R.M., Charo I.F., Jenne C.N., Kubes P. (2015). A dynamic spectrum of monocytes arising from the in situ reprogramming of CCR2+ monocytes at a site of sterile injury. J. Exp. Med..

[53] Fadok V.A., Voelker D.R., Campbell P.A., Cohen J.J., Bratton D.L., Henson P.M. (1992). Exposure of phosphatidylserine on the surface of apoptotic lymphocytes triggers specific recognition and removal by macrophages. J. Immunol..

[54] Segawa K., Yanagihashi Y., Yamada K., Suzuki C., Uchiyama Y., Nagata S. (2018). Phospholipid flippases enable precursor B cells to flee engulfment by macrophages. Proc. Natl. Acad. Sci. USA.

[55] Zargarian S., Shlomovitz I., Erlich Z., Hourizadeh A., Ofir-Birin Y., Croker B.A., Regev-Rudzki N., Edry-Botzer L., Gerlic M. (2017). Phosphatidylserine externalization, “necroptotic bodies” release, and phagocytosis during necroptosis. PLoS Biol..

[56] Ousingsawat J., Schreiber R., Kunzelmann K. (2019). TMEM16F/Anoctamin 6 in Ferroptotic Cell Death. Cancers.

[57] Wang Q., Imamura R., Motani K., Kushiyama H., Nagata S., Suda T. (2013). Pyroptotic cells externalize eat-me and release find-me signals and are efficiently engulfed by macrophages. Int. Immunol..

[58] Westman J., Grinstein S., Marques P.E. (2019). Phagocytosis of Necrotic Debris at Sites of Injury and Inflammation. Front. Immunol..

[59] Geng K., Kumar S., Kimani S.G., Kholodovych V., Kasikara C., Mizuno K., Sandiford O., Rameshwar P., Kotenko S.V., Birge R.B. (2017). Requirement of Gamma-Carboxyglutamic Acid Modification and Phosphatidylserine Binding for the Activation of Tyro3, Axl, and Mertk Receptors by Growth Arrest-Specific 6. Front. Immunol..

[60] Llacuna L., Barcena C., Bellido-Martin L., Fernandez L., Stefanovic M., Mari M., Garcia-Ruiz C., Fernandez-Checa J.C., Garcia de Frutos P., Morales A. (2010). Growth arrest-specific protein 6 is hepatoprotective against murine ischemia/reperfusion injury. Hepatology.

[61] Li Z., Weinman S.A. (2018). Regulation of Hepatic Inflammation via Macrophage Cell Death. Semin. Liver Dis..

[62] Bellan M., Cittone M.G., Tonello S., Rigamonti C., Castello L.M., Gavelli F., Pirisi M., Sainaghi P.P. (2019). Gas6/TAM System: A Key Modulator of the Interplay between Inflammation and Fibrosis. Int. J. Mol. Sci..

[63] Rantakari P., Patten D.A., Valtonen J., Karikoski M., Gerke H., Dawes H., Laurila J., Ohlmeier S., Elima K., Hubscher S.G. (2016). Stabilin-1 expression defines a subset of macrophages that mediate tissue homeostasis and prevent fibrosis in chronic liver injury. Proc. Natl. Acad. Sci. USA.

[64] Ge Y., Huang M., Yao Y.M. (2022). Efferocytosis and Its Role in Inflammatory Disorders. Front. Cell Dev. Biol..

[65] Oldenborg P.A., Zheleznyak A., Fang Y.F., Lagenaur C.F., Gresham H.D., Lindberg F.P. (2000). Role of CD47 as a marker of self on red blood cells. Science.

[66] Poon I.K., Lucas C.D., Rossi A.G., Ravichandran K.S. (2014). Apoptotic cell clearance: Basic biology and therapeutic potential. Nat. Rev. Immunol..

[67] Elward K., Griffiths M., Mizuno M., Harris C.L., Neal J.W., Morgan B.P., Gasque P. (2005). CD46 plays a key role in tailoring innate immune recognition of apoptotic and necrotic cells. J. Biol. Chem..

[68] Lerbs T., Cui L., King M.E., Chai T., Muscat C., Chung L., Brown R., Rieger K., Shibata T., Wernig G. (2020). CD47 prevents the elimination of diseased fibroblasts in scleroderma. JCI Insight.

[69] Shi H., Wang X., Li F., Gerlach B.D., Yurdagul A., Moore M.P., Zeldin S., Zhang H., Cai B., Zheng Z. (2022). CD47-SIRPalpha axis blockade in NASH promotes necroptotic hepatocyte clearance by liver macrophages and decreases hepatic fibrosis. Sci. Transl. Med..

[70] Park Y.J., Liu G., Lorne E.F., Zhao X., Wang J., Tsuruta Y., Zmijewski J., Abraham E. (2008). PAI-1 inhibits neutrophil efferocytosis. Proc. Natl. Acad. Sci. USA.

[71] Chidiac A.S., Buckley N.A., Noghrehchi F., Cairns R. (2023). Paracetamol (acetaminophen) overdose and hepatotoxicity: Mechanism, treatment, prevention measures, and estimates of burden of disease. Expert Opin. Drug Metab. Toxicol..

[72] Reuben A., Tillman H., Fontana R.J., Davern T., McGuire B., Stravitz R.T., Durkalski V., Larson A.M., Liou I., Fix O. (2016). Outcomes in Adults With Acute Liver Failure Between 1998 and 2013: An Observational Cohort Study. Ann. Intern. Med..

[73] Andrade R.J., Chalasani N., Bjornsson E.S., Suzuki A., Kullak-Ublick G.A., Watkins P.B., Devarbhavi H., Merz M., Lucena M.I., Kaplowitz N. (2019). Drug-induced liver injury. Nat. Rev. Dis. Primers.

[74] Iorga A., Dara L., Kaplowitz N. (2017). Drug-Induced Liver Injury: Cascade of Events Leading to Cell Death, Apoptosis or Necrosis. Int. J. Mol. Sci..

[75] Dara L., Johnson H., Suda J., Win S., Gaarde W., Han D., Kaplowitz N. (2015). Receptor interacting protein kinase 1 mediates murine acetaminophen toxicity independent of the necrosome and not through necroptosis. Hepatology.

[76] Kon K., Kim J.S., Jaeschke H., Lemasters J.J. (2004). Mitochondrial permeability transition in acetaminophen-induced necrosis and apoptosis of cultured mouse hepatocytes. Hepatology.

[77] Yan W., Shen Y., Huang J., Lu L., Zhang Q. (2021). MCC950 Ameliorates Acute Liver Injury Through Modulating Macrophage Polarization and Myeloid-Derived Suppressor Cells Function. Front. Med..

[78] Li Z., Wang H., Zhu J., Nan N., Lin Y., Zhuang X., Li L., Zhang Y., Huang P. (2022). Inhibition of TWEAK/Tnfrsf12a axis protects against acute liver failure by suppressing RIPK1-dependent apoptosis. Cell Death Discov..

[79] Yang C., Sun P., Deng M., Loughran P., Li W., Yi Z., Li S., Zhang X., Fan J., Billiar T.R. (2019). Gasdermin D protects against noninfectious liver injury by regulating apoptosis and necroptosis. Cell Death Dis..

[80] McGill M.R., Sharpe M.R., Williams C.D., Taha M., Curry S.C., Jaeschke H. (2012). The mechanism underlying acetaminophen-induced hepatotoxicity in humans and mice involves mitochondrial damage and nuclear DNA fragmentation. J. Clin. Investig..

[81] McGill M.R., Yan H.M., Ramachandran A., Murray G.J., Rollins D.E., Jaeschke H. (2011). HepaRG cells: A human model to study mechanisms of acetaminophen hepatotoxicity. Hepatology.

[82] Volkmann X., Anstaett M., Hadem J., Stiefel P., Bahr M.J., Lehner F., Manns M.P., Schulze-Osthoff K., Bantel H. (2008). Caspase activation is associated with spontaneous recovery from acute liver failure. Hepatology.

[83] Dhanasekaran D.N., Reddy E.P. (2008). JNK signaling in apoptosis. Oncogene.

[84] Xie Y., McGill M.R., Dorko K., Kumer S.C., Schmitt T.M., Forster J., Jaeschke H. (2014). Mechanisms of acetaminophen-induced cell death in primary human hepatocytes. Toxicol. Appl. Pharmacol..

[85] Yi Y., Zhang W., Tao L., Shao Q., Xu Q., Chen Y., Zhang H., Zhang J., Weng D. (2021). RIP1 kinase inactivation protects against acetaminophen-induced acute liver injury in mice. Free Radic. Biol. Med..

[86] Hameed H., Farooq M., Piquet-Pellorce C., Hamon A., Samson M., Le Seyec J. (2022). Questioning the RIPK1 kinase activity involvement in acetaminophen-induced hepatotoxicity in mouse. Free Radic. Biol. Med..

[87] Yamada N., Karasawa T., Kimura H., Watanabe S., Komada T., Kamata R., Sampilvanjil A., Ito J., Nakagawa K., Kuwata H. (2020). Ferroptosis driven by radical oxidation of n-6 polyunsaturated fatty acids mediates acetaminophen-induced acute liver failure. Cell Death Dis..

[88] Lorincz T., Jemnitz K., Kardon T., Mandl J., Szarka A. (2015). Ferroptosis is Involved in Acetaminophen Induced Cell Death. Pathol. Oncol. Res..

[89] Shan X., Li J., Liu J., Feng B., Zhang T., Liu Q., Ma H., Wu H., Wu H. (2023). Targeting ferroptosis by poly(acrylic) acid coated Mn_3_O_4_ nanoparticles alleviates acute liver injury. Nat. Commun..

[90] Ni H.M., Bockus A., Boggess N., Jaeschke H., Ding W.X. (2012). Activation of autophagy protects against acetaminophen-induced hepatotoxicity. Hepatology.

[91] Ron D., Walter P. (2007). Signal integration in the endoplasmic reticulum unfolded protein response. Nat. Rev. Mol. Cell Biol..

[92] Ye H., Chen C., Wu H., Zheng K., Martin-Adrados B., Caparros E., Frances R., Nelson L.J., Gomez Del Moral M., Asensio I. (2022). Genetic and pharmacological inhibition of XBP1 protects against APAP hepatotoxicity through the activation of autophagy. Cell Death Dis..

[93] Huang D.Q., Mathurin P., Cortez-Pinto H., Loomba R. (2023). Global epidemiology of alcohol-associated cirrhosis and HCC: Trends, projections and risk factors. Nat. Rev. Gastroenterol. Hepatol..

[94] Miyata T., Nagy L.E. (2020). Programmed cell death in alcohol-associated liver disease. Clin. Mol. Hepatol..

[95] Khanova E., Wu R., Wang W., Yan R., Chen Y., French S.W., Llorente C., Pan S.Q., Yang Q., Li Y. (2018). Pyroptosis by caspase11/4-gasdermin-D pathway in alcoholic hepatitis in mice and patients. Hepatology.

[96] Petrasek J., Iracheta-Vellve A., Saha B., Satishchandran A., Kodys K., Fitzgerald K.A., Kurt-Jones E.A., Szabo G. (2015). Metabolic danger signals, uric acid and ATP, mediate inflammatory cross-talk between hepatocytes and immune cells in alcoholic liver disease. J. Leukoc. Biol..

[97] Iracheta-Vellve A., Petrasek J., Satishchandran A., Gyongyosi B., Saha B., Kodys K., Fitzgerald K.A., Kurt-Jones E.A., Szabo G. (2015). Inhibition of sterile danger signals, uric acid and ATP, prevents inflammasome activation and protects from alcoholic steatohepatitis in mice. J. Hepatol..

[98] Heo M.J., Kim T.H., You J.S., Blaya D., Sancho-Bru P., Kim S.G. (2019). Alcohol dysregulates miR-148a in hepatocytes through FoxO1, facilitating pyroptosis via TXNIP overexpression. Gut.

[99] Nagata S., Tanaka M. (2017). Programmed cell death and the immune system. Nat. Rev. Immunol..

[100] Mandrekar P., Szabo G. (2009). Signalling pathways in alcohol-induced liver inflammation. J. Hepatol..

[101] Pan X.S., Li B.W., Wang L.L., Li N., Lin H.M., Zhang J., Du N., Zhu Y.Q., Wu X., Hu C.M. (2023). Kupffer cell pyroptosis mediated by METTL3 contributes to the progression of alcoholic steatohepatitis. FASEB J..

[102] Contreras-Zentella M.L., Villalobos-Garcia D., Hernandez-Munoz R. (2022). Ethanol Metabolism in the Liver, the Induction of Oxidant Stress, and the Antioxidant Defense System. Antioxidants.

[103] Venkatraman A., Shiva S., Wigley A., Ulasova E., Chhieng D., Bailey S.M., Darley-Usmar V.M. (2004). The role of iNOS in alcohol-dependent hepatotoxicity and mitochondrial dysfunction in mice. Hepatology.

[104] Ji C. (2014). New Insights into the Pathogenesis of Alcohol-Induced ER Stress and Liver Diseases. Int. J. Hepatol..

[105] Brentnall M., Rodriguez-Menocal L., De Guevara R.L., Cepero E., Boise L.H. (2013). Caspase-9, caspase-3 and caspase-7 have distinct roles during intrinsic apoptosis. BMC Cell Biol..

[106] Zeng T., Zhang C.L., Xiao M., Yang R., Xie K.Q. (2016). Critical Roles of Kupffer Cells in the Pathogenesis of Alcoholic Liver Disease: From Basic Science to Clinical Trials. Front. Immunol..

[107] Keshavarzian A., Farhadi A., Forsyth C.B., Rangan J., Jakate S., Shaikh M., Banan A., Fields J.Z. (2009). Evidence that chronic alcohol exposure promotes intestinal oxidative stress, intestinal hyperpermeability and endotoxemia prior to development of alcoholic steatohepatitis in rats. J. Hepatol..

[108] Kishore R., Hill J.R., McMullen M.R., Frenkel J., Nagy L.E. (2002). ERK1/2 and Egr-1 contribute to increased TNF-alpha production in rat Kupffer cells after chronic ethanol feeding. Am. J. Physiol. Gastrointest. Liver Physiol..

[109] Kolios G., Valatas V., Kouroumalis E. (2006). Role of Kupffer cells in the pathogenesis of liver disease. World J. Gastroenterol..

[110] Parlesak A., Schafer C., Schutz T., Bode J.C., Bode C. (2000). Increased intestinal permeability to macromolecules and endotoxemia in patients with chronic alcohol abuse in different stages of alcohol-induced liver disease. J. Hepatol..

[111] Roychowdhury S., Chiang D.J., Mandal P., McMullen M.R., Liu X., Cohen J.I., Pollard J., Feldstein A.E., Nagy L.E. (2012). Inhibition of apoptosis protects mice from ethanol-mediated acceleration of early markers of CCl4-induced fibrosis but not steatosis or inflammation. Alcohol. Clin. Exp. Res..

[112] Roychowdhury S., McMullen M.R., Pisano S.G., Liu X., Nagy L.E. (2013). Absence of receptor interacting protein kinase 3 prevents ethanol-induced liver injury. Hepatology.

[113] Miyata T., Wu X., Fan X., Huang E., Sanz-Garcia C., Ross C.K.C., Roychowdhury S., Bellar A., McMullen M.R., Dasarathy J. (2021). Differential role of MLKL in alcohol-associated and non-alcohol-associated fatty liver diseases in mice and humans. JCI Insight.

[114] Wu X., Fan X., McMullen M.R., Miyata T., Kim A., Pathak V., Wu J., Day L.Z., Hardesty J.E., Welch N. (2023). Macrophage-derived MLKL in alcohol-associated liver disease: Regulation of phagocytosis. Hepatology.

[115] Liu C.Y., Wang M., Yu H.M., Han F.X., Wu Q.S., Cai X.J., Kurihara H., Chen Y.X., Li Y.F., He R.R. (2020). Ferroptosis is involved in alcohol-induced cell death in vivo and in vitro. Biosci. Biotechnol. Biochem..

[116] Zhang Y., Zhao S., Fu Y., Yan L., Feng Y., Chen Y., Wu Y., Deng Y., Zhang G., Chen Z. (2020). Computational repositioning of dimethyl fumarate for treating alcoholic liver disease. Cell Death Dis..

[117] Zhou Z., Ye T.J., DeCaro E., Buehler B., Stahl Z., Bonavita G., Daniels M., You M. (2020). Intestinal SIRT1 Deficiency Protects Mice from Ethanol-Induced Liver Injury by Mitigating Ferroptosis. Am. J. Pathol..

[118] Zhou Z., Ye T.J., Bonavita G., Daniels M., Kainrad N., Jogasuria A., You M. (2019). Adipose-Specific Lipin-1 Overexpression Renders Hepatic Ferroptosis and Exacerbates Alcoholic Steatohepatitis in Mice. Hepatol. Commun..

[119] Ding W.X., Li M., Chen X., Ni H.M., Lin C.W., Gao W., Lu B., Stolz D.B., Clemens D.L., Yin X.M. (2010). Autophagy reduces acute ethanol-induced hepatotoxicity and steatosis in mice. Gastroenterology.

[120] Rautou P.E., Mansouri A., Lebrec D., Durand F., Valla D., Moreau R. (2010). Autophagy in liver diseases. J. Hepatol..

[121] Thomes P.G., Trambly C.S., Fox H.S., Tuma D.J., Donohue T.M. (2015). Acute and Chronic Ethanol Administration Differentially Modulate Hepatic Autophagy and Transcription Factor EB. Alcohol. Clin. Exp. Res..

[122] Chao X., Wang S., Zhao K., Li Y., Williams J.A., Li T., Chavan H., Krishnamurthy P., He X.C., Li L. (2018). Impaired TFEB-Mediated Lysosome Biogenesis and Autophagy Promote Chronic Ethanol-Induced Liver Injury and Steatosis in Mice. Gastroenterology.

[123] Kucukoglu O., Guldiken N., Chen Y., Usachov V., El-Heliebi A., Haybaeck J., Denk H., Trautwein C., Strnad P. (2014). High-fat diet triggers Mallory-Denk body formation through misfolding and crosslinking of excess keratin 8. Hepatology.

[124] Watanabe A., Sohail M.A., Gomes D.A., Hashmi A., Nagata J., Sutterwala F.S., Mahmood S., Jhandier M.N., Shi Y., Flavell R.A. (2009). Inflammasome-mediated regulation of hepatic stellate cells. Am. J. Physiol. Gastrointest. Liver Physiol..

[125] Gurung P., Lukens J.R., Kanneganti T.D. (2015). Mitochondria: Diversity in the regulation of the NLRP3 inflammasome. Trends Mol. Med..

[126] Gaul S., Leszczynska A., Alegre F., Kaufmann B., Johnson C.D., Adams L.A., Wree A., Damm G., Seehofer D., Calvente C.J. (2021). Hepatocyte pyroptosis and release of inflammasome particles induce stellate cell activation and liver fibrosis. J. Hepatol..

[127] Xu B., Jiang M., Chu Y., Wang W., Chen D., Li X., Zhang Z., Zhang D., Fan D., Nie Y. (2018). Gasdermin D plays a key role as a pyroptosis executor of non-alcoholic steatohepatitis in humans and mice. J. Hepatol..

[128] Kaufmann B., Kui L., Reca A., Leszczynska A., Kim A.D., Booshehri L.M., Wree A., Friess H., Hartmann D., Broderick L. (2022). Cell-specific Deletion of NLRP3 Inflammasome Identifies Myeloid Cells as Key Drivers of Liver Inflammation and Fibrosis in Murine Steatohepatitis. Cell. Mol. Gastroenterol. Hepatol..

[129] Feldstein A.E., Canbay A., Angulo P., Taniai M., Burgart L.J., Lindor K.D., Gores G.J. (2003). Hepatocyte apoptosis and fas expression are prominent features of human nonalcoholic steatohepatitis. Gastroenterology.

[130] Ferreira D.M., Castro R.E., Machado M.V., Evangelista T., Silvestre A., Costa A., Coutinho J., Carepa F., Cortez-Pinto H., Rodrigues C.M. (2011). Apoptosis and insulin resistance in liver and peripheral tissues of morbidly obese patients is associated with different stages of non-alcoholic fatty liver disease. Diabetologia.

[131] Tenney J.R., Rozhkov L., Horn P., Miles L., Miles M.V. (2014). Cerebral glucose hypometabolism is associated with mitochondrial dysfunction in patients with intractable epilepsy and cortical dysplasia. Epilepsia.

[132] Machado M.V., Michelotti G.A., Pereira T.d.A., Boursier J., Kruger L., Swiderska-Syn M., Karaca G., Xie G., Guy C.D., Bohinc B. (2015). Reduced lipoapoptosis, hedgehog pathway activation and fibrosis in caspase-2 deficient mice with non-alcoholic steatohepatitis. Gut.

[133] Thapaliya S., Wree A., Povero D., Inzaugarat M.E., Berk M., Dixon L., Papouchado B.G., Feldstein A.E. (2014). Caspase 3 inactivation protects against hepatic cell death and ameliorates fibrogenesis in a diet-induced NASH model. Dig. Dis. Sci..

[134] Zhao P., Sun X., Chaggan C., Liao Z., In Wong K., He F., Singh S., Loomba R., Karin M., Witztum J.L. (2020). An AMPK–caspase-6 axis controls liver damage in nonalcoholic steatohepatitis. Science.

[135] Harrison S.A., Goodman Z., Jabbar A., Vemulapalli R., Younes Z.H., Freilich B., Sheikh M.Y., Schattenberg J.M., Kayali Z., Zivony A. (2020). A randomized, placebo-controlled trial of emricasan in patients with NASH and F1-F3 fibrosis. J. Hepatol..

[136] Tiegs G., Horst A.K. (2022). TNF in the liver: Targeting a central player in inflammation. Semin. Immunopathol..

[137] Afonso M.B., Rodrigues P.M., Mateus-Pinheiro M., Simao A.L., Gaspar M.M., Majdi A., Arretxe E., Alonso C., Santos-Laso A., Jimenez-Aguero R. (2021). RIPK3 acts as a lipid metabolism regulator contributing to inflammation and carcinogenesis in non-alcoholic fatty liver disease. Gut.

[138] Chenxu G., Minxuan X., Yuting Q., Tingting G., Jing F., Jinxiao L., Sujun W., Yongjie M., Deshuai L., Qiang L. (2019). Loss of RIP3 initiates annihilation of high-fat diet initialized nonalcoholic hepatosteatosis: A mechanism involving Toll-like receptor 4 and oxidative stress. Free Radic. Biol. Med..

[139] Ren Y., Su Y., Sun L., He S., Meng L., Liao D., Liu X., Ma Y., Liu C., Li S. (2017). Discovery of a Highly Potent, Selective, and Metabolically Stable Inhibitor of Receptor-Interacting Protein 1 (RIP1) for the Treatment of Systemic Inflammatory Response Syndrome. J. Med. Chem..

[140] Wu X., Poulsen K.L., Sanz-Garcia C., Huang E., McMullen M.R., Roychowdhury S., Dasarathy S., Nagy L.E. (2020). MLKL-dependent signaling regulates autophagic flux in a murine model of non-alcohol-associated fatty liver and steatohepatitis. J. Hepatol..

[141] Kowdley K.V., Belt P., Wilson L.A., Yeh M.M., Neuschwander-Tetri B.A., Chalasani N., Sanyal A.J., Nelson J.E., the NASH Clinical Research Network (2012). Serum ferritin is an independent predictor of histologic severity and advanced fibrosis in patients with nonalcoholic fatty liver disease. Hepatology.

[142] Gao G., Xie Z., Li E.W., Yuan Y., Fu Y., Wang P., Zhang X., Qiao Y., Xu J., Holscher C. (2021). Dehydroabietic acid improves nonalcoholic fatty liver disease through activating the Keap1/Nrf2-ARE signaling pathway to reduce ferroptosis. J. Nat. Med..

[143] Loguercio C., De Girolamo V., de Sio I., Tuccillo C., Ascione A., Baldi F., Budillon G., Cimino L., Di Carlo A., Di Marino M.P. (2001). Non-alcoholic fatty liver disease in an area of southern Italy: Main clinical, histological, and pathophysiological aspects. J. Hepatol..

[144] Wei S., Qiu T., Wang N., Yao X., Jiang L., Jia X., Tao Y., Zhang J., Zhu Y., Yang G. (2020). Ferroptosis mediated by the interaction between Mfn2 and IREalpha promotes arsenic-induced nonalcoholic steatohepatitis. Environ. Res..

[145] Qi J., Kim J.W., Zhou Z., Lim C.W., Kim B. (2020). Ferroptosis Affects the Progression of Nonalcoholic Steatohepatitis via the Modulation of Lipid Peroxidation-Mediated Cell Death in Mice. Am. J. Pathol..

[146] Li X., Wang T.X., Huang X., Li Y., Sun T., Zang S., Guan K.L., Xiong Y., Liu J., Yuan H.X. (2020). Targeting ferroptosis alleviates methionine-choline deficient (MCD)-diet induced NASH by suppressing liver lipotoxicity. Liver Int..

[147] Gonzalez-Rodriguez A., Mayoral R., Agra N., Valdecantos M.P., Pardo V., Miquilena-Colina M.E., Vargas-Castrillon J., Lo Iacono O., Corazzari M., Fimia G.M. (2014). Impaired autophagic flux is associated with increased endoplasmic reticulum stress during the development of NAFLD. Cell Death Dis..

[148] Rodriguez-Navarro J.A., Kaushik S., Koga H., Dall’Armi C., Shui G., Wenk M.R., Di Paolo G., Cuervo A.M. (2012). Inhibitory effect of dietary lipids on chaperone-mediated autophagy. Proc. Natl. Acad. Sci. USA.

[149] Yang L., Li P., Fu S., Calay E.S., Hotamisligil G.S. (2010). Defective hepatic autophagy in obesity promotes ER stress and causes insulin resistance. Cell Metab..

[150] Wen C.P., Lin J., Yang Y.C., Tsai M.K., Tsao C.K., Etzel C., Huang M., Hsu C.Y., Ye Y., Mishra L. (2012). Hepatocellular carcinoma risk prediction model for the general population: The predictive power of transaminases. J. Natl. Cancer Inst..

[151] Moossavi M., Parsamanesh N., Bahrami A., Atkin S.L., Sahebkar A. (2018). Role of the NLRP3 inflammasome in cancer. Mol. Cancer.

[152] Jiang M., Qi L., Li L., Li Y. (2020). The caspase-3/GSDME signal pathway as a switch between apoptosis and pyroptosis in cancer. Cell Death Discov..

[153] Xia X., Wang X., Cheng Z., Qin W., Lei L., Jiang J., Hu J. (2019). The role of pyroptosis in cancer: Pro-cancer or pro-”host”?. Cell Death Dis..

[154] Fang G., Zhang Q., Fan J., Li H., Ding Z., Fu J., Wu Y., Zeng Y., Liu J. (2022). Pyroptosis related genes signature predicts prognosis and immune infiltration of tumor microenvironment in hepatocellular carcinoma. BMC Cancer.

[155] Li Y., Li Y., Zhang X., Duan X., Feng H., Yu Z., Gao Y. (2022). A novel association of pyroptosis-related gene signature with the prognosis of hepatocellular carcinoma. Front. Oncol..

[156] Chu Q., Jiang Y., Zhang W., Xu C., Du W., Tuguzbaeva G., Qin Y., Li A., Zhang L., Sun G. (2016). Pyroptosis is involved in the pathogenesis of human hepatocellular carcinoma. Oncotarget.

[157] Wei Q., Mu K., Li T., Zhang Y., Yang Z., Jia X., Zhao W., Huai W., Guo P., Han L. (2014). Deregulation of the NLRP3 inflammasome in hepatic parenchymal cells during liver cancer progression. Lab. Investig..

[158] Yan Z., Da Q., Li Z., Lin Q., Yi J., Su Y., Yu G., Ren Q., Liu X., Lin Z. (2022). Inhibition of NEK7 Suppressed Hepatocellular Carcinoma Progression by Mediating Cancer Cell Pyroptosis. Front. Oncol..

[159] Weber A., Boger R., Vick B., Urbanik T., Haybaeck J., Zoller S., Teufel A., Krammer P.H., Opferman J.T., Galle P.R. (2010). Hepatocyte-specific deletion of the antiapoptotic protein myeloid cell leukemia-1 triggers proliferation and hepatocarcinogenesis in mice. Hepatology.

[160] Hikita H., Kodama T., Shimizu S., Li W., Shigekawa M., Tanaka S., Hosui A., Miyagi T., Tatsumi T., Kanto T. (2012). Bak deficiency inhibits liver carcinogenesis: A causal link between apoptosis and carcinogenesis. J. Hepatol..

[161] Schneider A.T., Gautheron J., Feoktistova M., Roderburg C., Loosen S.H., Roy S., Benz F., Schemmer P., Buchler M.W., Nachbur U. (2017). RIPK1 Suppresses a TRAF2-Dependent Pathway to Liver Cancer. Cancer Cell.

[162] Tan S., Zhao J., Sun Z., Cao S., Niu K., Zhong Y., Wang H., Shi L., Pan H., Hu J. (2020). Hepatocyte-specific TAK1 deficiency drives RIPK1 kinase-dependent inflammation to promote liver fibrosis and hepatocellular carcinoma. Proc. Natl. Acad. Sci. USA.

[163] Mohammed S., Thadathil N., Ohene-Marfo P., Tran A.L., Van Der Veldt M., Georgescu C., Oh S., Nicklas E.H., Wang D., Haritha N.H. (2023). Absence of Either Ripk3 or Mlkl Reduces Incidence of Hepatocellular Carcinoma Independent of Liver Fibrosis. Mol. Cancer Res..

[164] Vucur M., Ghallab A., Schneider A.T., Adili A., Cheng M., Castoldi M., Singer M.T., Buttner V., Keysberg L.S., Kusgens L. (2023). Sublethal necroptosis signaling promotes inflammation and liver cancer. Immunity.

[165] Li J., Tang M., Ke R.X., Li P.L., Sheng Z.G., Zhu B.Z. (2024). The anti-cancer drug candidate CBL0137 induced necroptosis via forming left-handed Z-DNA and its binding protein ZBP1 in liver cells. Toxicol. Appl. Pharmacol..

[166] Tang B., Zhu J., Li J., Fan K., Gao Y., Cheng S., Kong C., Zheng L., Wu F., Weng Q. (2020). The ferroptosis and iron-metabolism signature robustly predicts clinical diagnosis, prognosis and immune microenvironment for hepatocellular carcinoma. Cell Commun. Signal..

[167] Liang J.Y., Wang D.S., Lin H.C., Chen X.X., Yang H., Zheng Y., Li Y.H. (2020). A Novel Ferroptosis-related Gene Signature for Overall Survival Prediction in Patients with Hepatocellular Carcinoma. Int. J. Biol. Sci..

[168] He F., Zhang P., Liu J., Wang R., Kaufman R.J., Yaden B.C., Karin M. (2023). ATF4 suppresses hepatocarcinogenesis by inducing SLC7A11 (xCT) to block stress-related ferroptosis. J. Hepatol..

[169] Sun X., Ou Z., Chen R., Niu X., Chen D., Kang R., Tang D. (2016). Activation of the p62-Keap1-NRF2 pathway protects against ferroptosis in hepatocellular carcinoma cells. Hepatology.

[170] Jiang L., Kon N., Li T., Wang S.J., Su T., Hibshoosh H., Baer R., Gu W. (2015). Ferroptosis as a p53-mediated activity during tumour suppression. Nature.

[171] Wu K., Yan M., Liu T., Wang Z., Duan Y., Xia Y., Ji G., Shen Y., Wang L., Li L. (2023). Creatine kinase B suppresses ferroptosis by phosphorylating GPX4 through a moonlighting function. Nat. Cell Biol..

[172] White E. (2015). The role for autophagy in cancer. J. Clin. Investig..

[173] Zatloukal K., Stumptner C., Fuchsbichler A., Heid H., Schnoelzer M., Kenner L., Kleinert R., Prinz M., Aguzzi A., Denk H. (2002). p62 Is a common component of cytoplasmic inclusions in protein aggregation diseases. Am. J. Pathol..

[174] Umemura A., He F., Taniguchi K., Nakagawa H., Yamachika S., Font-Burgada J., Zhong Z., Subramaniam S., Raghunandan S., Duran A. (2016). p62, Upregulated during Preneoplasia, Induces Hepatocellular Carcinogenesis by Maintaining Survival of Stressed HCC-Initiating Cells. Cancer Cell.

[175] Desideri E., Castelli S., Dorard C., Toifl S., Grazi G.L., Ciriolo M.R., Baccarini M. (2023). Impaired degradation of YAP1 and IL6ST by chaperone-mediated autophagy promotes proliferation and migration of normal and hepatocellular carcinoma cells. Autophagy.

[176] Ueno T., Sato W., Horie Y., Komatsu M., Tanida I., Yoshida M., Ohshima S., Mak T.W., Watanabe S., Kominami E. (2008). Loss of Pten, a tumor suppressor, causes the strong inhibition of autophagy without affecting LC3 lipidation. Autophagy.

[177] Thoen L.F., Guimaraes E.L., Dolle L., Mannaerts I., Najimi M., Sokal E., van Grunsven L.A. (2011). A role for autophagy during hepatic stellate cell activation. J. Hepatol..

[178] Myojin Y., Hikita H., Sugiyama M., Sasaki Y., Fukumoto K., Sakane S., Makino Y., Takemura N., Yamada R., Shigekawa M. (2021). Hepatic Stellate Cells in Hepatocellular Carcinoma Promote Tumor Growth Via Growth Differentiation Factor 15 Production. Gastroenterology.

[179] Duran A., Hernandez E.D., Reina-Campos M., Castilla E.A., Subramaniam S., Raghunandan S., Roberts L.R., Kisseleva T., Karin M., Diaz-Meco M.T. (2016). p62/SQSTM1 by Binding to Vitamin D Receptor Inhibits Hepatic Stellate Cell Activity, Fibrosis, and Liver Cancer. Cancer Cell.

[180] Donne R., Lujambio A. (2023). The liver cancer immune microenvironment: Therapeutic implications for hepatocellular carcinoma. Hepatology.

[181] Cheng H.Y., Hsieh C.H., Lin P.H., Chen Y.T., Hsu D.S., Tai S.K., Chu P.Y., Yang M.H. (2022). Snail-regulated exosomal microRNA-21 suppresses NLRP3 inflammasome activity to enhance cisplatin resistance. J. Immunother. Cancer.

[182] Ershaid N., Sharon Y., Doron H., Raz Y., Shani O., Cohen N., Monteran L., Leider-Trejo L., Ben-Shmuel A., Yassin M. (2019). NLRP3 inflammasome in fibroblasts links tissue damage with inflammation in breast cancer progression and metastasis. Nat. Commun..

[183] Hu Y., Chen D., Hong M., Liu J., Li Y., Hao J., Lu L., Yin Z., Wu Y. (2022). Apoptosis, Pyroptosis, and Ferroptosis Conspiringly Induce Immunosuppressive Hepatocellular Carcinoma Microenvironment and gammadelta T-Cell Imbalance. Front. Immunol..

[184] Song Y., Kim N., Heo J., Shum D., Heo T., Seo H.R. (2024). Inhibition of DNMT3B expression in activated hepatic stellate cells overcomes chemoresistance in the tumor microenvironment of hepatocellular carcinoma. Sci. Rep..

[185] Wang C., Chen C., Hu W., Tao L., Chen J. (2023). Revealing the role of necroptosis microenvironment: FCGBP + tumor-associated macrophages drive primary liver cancer differentiation towards cHCC-CCA or iCCA. Apoptosis.

[186] Seehawer M., Heinzmann F., D’Artista L., Harbig J., Roux P.F., Hoenicke L., Dang H., Klotz S., Robinson L., Dore G. (2018). Necroptosis microenvironment directs lineage commitment in liver cancer. Nature.

[187] Conche C., Finkelmeier F., Pesic M., Nicolas A.M., Bottger T.W., Kennel K.B., Denk D., Ceteci F., Mohs K., Engel E. (2023). Combining ferroptosis induction with MDSC blockade renders primary tumours and metastases in liver sensitive to immune checkpoint blockade. Gut.

[188] Song Y.J., Zhang S.S., Guo X.L., Sun K., Han Z.P., Li R., Zhao Q.D., Deng W.J., Xie X.Q., Zhang J.W. (2013). Autophagy contributes to the survival of CD133+ liver cancer stem cells in the hypoxic and nutrient-deprived tumor microenvironment. Cancer Lett..

[189] Lin C.W., Chen Y.S., Lin C.C., Lee P.H., Lo G.H., Hsu C.C., Hsieh P.M., Koh K.W., Chou T.C., Dai C.Y. (2018). Autophagy-related gene LC3 expression in tumor and liver microenvironments significantly predicts recurrence of hepatocellular carcinoma after surgical resection. Clin. Transl. Gastroenterol..

[190] Dar W.A., Sullivan E., Bynon J.S., Eltzschig H., Ju C. (2019). Ischaemia reperfusion injury in liver transplantation: Cellular and molecular mechanisms. Liver Int..

[191] Peralta C., Jimenez-Castro M.B., Gracia-Sancho J. (2013). Hepatic ischemia and reperfusion injury: Effects on the liver sinusoidal milieu. J. Hepatol..

[192] Bahde R., Spiegel H.U. (2010). Hepatic ischaemia-reperfusion injury from bench to bedside. Br. J. Surg..

[193] Ni H., Ou Z., Wang Y., Liu Y., Sun K., Zhang J., Zhang J., Deng W., Zeng W., Xia R. (2023). XBP1 modulates endoplasmic reticulum and mitochondria crosstalk via regulating NLRP3 in renal ischemia/reperfusion injury. Cell Death Discov..

[194] Xu L., Zeng Z., Niu C., Liu D., Lin S., Liu X., Szabo G., Lu J., Zheng S., Zhou P. (2023). Normothermic ex vivo heart perfusion with NLRP3 inflammasome inhibitor Mcc950 treatment improves cardiac function of circulatory death hearts after transplantation. Front. Cardiovasc. Med..

[195] Sadowsky D., Zamora R., Barclay D., Yin J., Fontes P., Vodovotz Y. (2016). Machine Perfusion of Porcine Livers with Oxygen-Carrying Solution Results in Reprogramming of Dynamic Inflammation Networks. Front. Pharmacol..

[196] He W., Ye S., Zeng C., Xue S., Hu X., Zhang X., Gao S., Xiong Y., He X., Vivalda S. (2018). Hypothermic oxygenated perfusion (HOPE) attenuates ischemia/reperfusion injury in the liver through inhibition of the TXNIP/NLRP3 inflammasome pathway in a rat model of donation after cardiac death. FASEB J..

[197] Scheuermann U., Zhu M., Song M., Yerxa J., Gao Q., Davis R.P., Zhang M., Parker W., Hartwig M.G., Kwun J. (2019). Damage-Associated Molecular Patterns Induce Inflammatory Injury During Machine Preservation of the Liver: Potential Targets to Enhance a Promising Technology. Liver Transplant..

[198] Yu Y., Cheng Y., Pan Q., Zhang Y.J., Jia D.G., Liu Y.F. (2019). Effect of the Selective NLRP3 Inflammasome Inhibitor mcc950 on Transplantation Outcome in a Pig Liver Transplantation Model With Organs From Donors After Circulatory Death Preserved by Hypothermic Machine Perfusion. Transplantation.

[199] Liu Y., Li S., Zhang G., Cai J. (2022). NOD1 induces pyroptotic cell death to aggravate liver ischemia-reperfusion injury in mice. MedComm.

[200] Kolachala V.L., Lopez C., Shen M., Shayakhmetov D., Gupta N.A. (2021). Ischemia reperfusion injury induces pyroptosis and mediates injury in steatotic liver thorough Caspase 1 activation. Apoptosis.

[201] Hu Y., Zhan F., Wang Y., Wang D., Lu H., Wu C., Xia Y., Meng L., Zhang F., Wang X. (2023). The Ninj1/Dusp1 Axis Contributes to Liver Ischemia Reperfusion Injury by Regulating Macrophage Activation and Neutrophil Infiltration. Cell Mol. Gastroenterol. Hepatol..

[202] Gao W., Bentley R.C., Madden J.F., Clavien P.A. (1998). Apoptosis of sinusoidal endothelial cells is a critical mechanism of preservation injury in rat liver transplantation. Hepatology.

[203] Sasaki H., Matsuno T., Tanaka N., Orita K. (1996). Activation of apoptosis during the reperfusion phase after rat liver ischemia. Transplant. Proc..

[204] Ye L., He S., Mao X., Zhang Y., Cai Y., Li S. (2020). Effect of Hepatic Macrophage Polarization and Apoptosis on Liver Ischemia and Reperfusion Injury During Liver Transplantation. Front. Immunol..

[205] Martin-Villalba A., Llorens-Bobadilla E., Wollny D. (2013). CD95 in cancer: Tool or target?. Trends Mol. Med..

[206] Al-Saeedi M., Steinebrunner N., Kudsi H., Halama N., Mogler C., Buchler M.W., Krammer P.H., Schemmer P., Muller M. (2018). Neutralization of CD95 ligand protects the liver against ischemia-reperfusion injury and prevents acute liver failure. Cell Death Dis..

[207] Wu S., Yang J., Sun G., Hu J., Zhang Q., Cai J., Yuan D., Li H., Hei Z., Yao W. (2021). Macrophage extracellular traps aggravate iron overload-related liver ischaemia/reperfusion injury. Br. J. Pharmacol..

[208] Saeed W.K., Jun D.W., Jang K., Chae Y.J., Lee J.S., Kang H.T. (2017). Does necroptosis have a crucial role in hepatic ischemia-reperfusion injury?. PLoS ONE.

[209] Rosentreter D., Funken D., Reifart J., Mende K., Rentsch M., Khandoga A. (2015). RIP1-Dependent Programmed Necrosis is Negatively Regulated by Caspases During Hepatic Ischemia-Reperfusion. Shock.

[210] Shi S., Bonaccorsi-Riani E., Schurink I., van den Bosch T., Doukas M., Lila K.A., Roest H.P., Xhema D., Gianello P., de Jonge J. (2022). Liver Ischemia and Reperfusion Induce Periportal Expression of Necroptosis Executor pMLKL Which Is Associated With Early Allograft Dysfunction After Transplantation. Front. Immunol..

[211] Ni H.M., Chao X., Kaseff J., Deng F., Wang S., Shi Y.H., Li T., Ding W.X., Jaeschke H. (2019). Receptor-Interacting Serine/Threonine-Protein Kinase 3 (RIPK3)-Mixed Lineage Kinase Domain-Like Protein (MLKL)-Mediated Necroptosis Contributes to Ischemia-Reperfusion Injury of Steatotic Livers. Am. J. Pathol..

[212] Baidya R., Gautheron J., Crawford D.H.G., Wang H., Bridle K.R. (2021). Inhibition of MLKL Attenuates Necroptotic Cell Death in a Murine Cell Model of Ischaemia Injury. J. Clin. Med..

[213] Zhong W., Wang X., Rao Z., Pan X., Sun Y., Jiang T., Wang P., Zhou H., Wang X. (2020). Aging aggravated liver ischemia and reperfusion injury by promoting hepatocyte necroptosis in an endoplasmic reticulum stress-dependent manner. Ann. Transl. Med..

[214] Xu J., Wu D., Zhou S., Hu H., Li F., Guan Z., Zhan X., Gao Y., Wang P., Rao Z. (2023). MLKL deficiency attenuated hepatocyte oxidative DNA damage by activating mitophagy to suppress macrophage cGAS-STING signaling during liver ischemia and reperfusion injury. Cell Death Discov..

[215] Xu D., Qu X., Tian Y., Jie Z., Xi Z., Xue F., Ma X., Zhu J., Xia Q. (2022). Macrophage Notch1 inhibits TAK1 function and RIPK3-mediated hepatocyte necroptosis through activation of beta-catenin signaling in liver ischemia and reperfusion injury. Cell Commun. Signal.

[216] Guo J., Song Z., Yu J., Li C., Jin C., Duan W., Liu X., Liu Y., Huang S., Tuo Y. (2022). Hepatocyte-specific TMEM16A deficiency alleviates hepatic ischemia/reperfusion injury via suppressing GPX4-mediated ferroptosis. Cell Death Dis..

[217] Morishita H., Mizushima N. (2019). Diverse Cellular Roles of Autophagy. Annu. Rev. Cell Dev. Biol..

[218] Liu A., Wang W., Lu Z., Liu Z., Zhou W., Zhong Z., Ye Q. (2021). Mild hypothermia pretreatment extenuates liver ischemia-reperfusion injury through Rab7-mediated autophagosomes-lysosomes fusion. Biochem. Biophys. Res. Commun..

[219] Kong W.N., Li W., Bai C., Dong Y., Wu Y., An W. (2022). Augmenter of liver regeneration-mediated mitophagy protects against hepatic ischemia/reperfusion injury. Am. J. Transplant..

[220] Witek R.P., Stone W.C., Karaca F.G., Syn W.K., Pereira T.A., Agboola K.M., Omenetti A., Jung Y., Teaberry V., Choi S.S. (2009). Pan-caspase inhibitor VX-166 reduces fibrosis in an animal model of nonalcoholic steatohepatitis. Hepatology.

[221] Anstee Q.M., Concas D., Kudo H., Levene A., Pollard J., Charlton P., Thomas H.C., Thursz M.R., Goldin R.D. (2010). Impact of pan-caspase inhibition in animal models of established steatosis and non-alcoholic steatohepatitis. J. Hepatol..

[222] Barreyro F.J., Holod S., Finocchietto P.V., Camino A.M., Aquino J.B., Avagnina A., Carreras M.C., Poderoso J.J., Gores G.J. (2015). The pan-caspase inhibitor Emricasan (IDN-6556) decreases liver injury and fibrosis in a murine model of non-alcoholic steatohepatitis. Liver Int..

[223] Frenette C.T., Morelli G., Shiffman M.L., Frederick R.T., Rubin R.A., Fallon M.B., Cheng J.T., Cave M., Khaderi S.A., Massoud O. (2019). Emricasan Improves Liver Function in Patients with Cirrhosis and High Model for End-Stage Liver Disease Scores Compared With Placebo. Clin. Gastroenterol. Hepatol..

[224] Ratziu V., Sheikh M.Y., Sanyal A.J., Lim J.K., Conjeevaram H., Chalasani N., Abdelmalek M., Bakken A., Renou C., Palmer M. (2012). A phase 2, randomized, double-blind, placebo-controlled study of GS-9450 in subjects with nonalcoholic steatohepatitis. Hepatology.

[225] Garcia-Tsao G., Bosch J., Kayali Z., Harrison S.A., Abdelmalek M.F., Lawitz E., Satapathy S.K., Ghabril M., Shiffman M.L., Younes Z.H. (2020). Randomized placebo-controlled trial of emricasan for non-alcoholic steatohepatitis-related cirrhosis with severe portal hypertension. J. Hepatol..

[226] Safadi R., Konikoff F.M., Mahamid M., Zelber-Sagi S., Halpern M., Gilat T., Oren R., FLORA Group (2014). The fatty acid-bile acid conjugate Aramchol reduces liver fat content in patients with nonalcoholic fatty liver disease. Clin. Gastroenterol. Hepatol..

[227] Huang F., Wang J., Yu F., Tang Y., Ding G., Yang Z., Sun Y. (2018). Protective Effect of Meretrix meretrix Oligopeptides on High-Fat-Diet-Induced Non-Alcoholic Fatty Liver Disease in Mice. Mar. Drugs.

[228] Choi Y.J., Johnson J.D., Lee J.J., Song J., Matthews M., Hellerstein M.K., McWherter C.A. (2024). Seladelpar combined with complementary therapies improves fibrosis, inflammation, and liver injury in a mouse model of nonalcoholic steatohepatitis. Am. J. Physiol. Gastrointest. Liver Physiol..

[229] Hajighasem A., Farzanegi P., Mazaheri Z. (2019). Effects of combined therapy with resveratrol, continuous and interval exercises on apoptosis, oxidative stress, and inflammatory biomarkers in the liver of old rats with non-alcoholic fatty liver disease. Arch. Physiol. Biochem..

[230] Sharma R.S., Harrison D.J., Kisielewski D., Cassidy D.M., McNeilly A.D., Gallagher J.R., Walsh S.V., Honda T., McCrimmon R.J., Dinkova-Kostova A.T. (2018). Experimental Nonalcoholic Steatohepatitis and Liver Fibrosis Are Ameliorated by Pharmacologic Activation of Nrf2 (NF-E2 p45-Related Factor 2). Cell. Mol. Gastroenterol. Hepatol..

[231] Park H.S., Song J.W., Park J.H., Lim B.K., Moon O.S., Son H.Y., Lee J.H., Gao B., Won Y.S., Kwon H.J. (2021). TXNIP/VDUP1 attenuates steatohepatitis via autophagy and fatty acid oxidation. Autophagy.

[232] Love S., Mudasir M.A., Bhardwaj S.C., Singh G., Tasduq S.A. (2017). Long-term administration of tacrolimus and everolimus prevents high cholesterol-high fructose-induced steatosis in C57BL/6J mice by inhibiting de-novo lipogenesis. Oncotarget.

[233] Lee S.G., Jeng L.-B., Saliba F., Soin A.S., Lee W.-C., De Simone P., Nevens F., Suh K.-S., Fischer L., Joo D.J. (2021). Efficacy and Safety of Everolimus With Reduced Tacrolimus in Liver Transplant Recipients: 24-month Results From the Pooled Analysis of 2 Randomized Controlled Trials. Transplantation.

[234] Zhang D., Ma Y., Liu J., Deng Y., Zhou B., Wen Y., Li M., Wen D., Ying Y., Luo S. (2021). Metformin Alleviates Hepatic Steatosis and Insulin Resistance in a Mouse Model of High-Fat Diet-Induced Nonalcoholic Fatty Liver Disease by Promoting Transcription Factor EB-Dependent Autophagy. Front. Pharmacol..

[235] Lu X., Xuan W., Li J., Yao H., Huang C., Li J. (2021). AMPK protects against alcohol-induced liver injury through UQCRC2 to up-regulate mitophagy. Autophagy.

[236] Kim S.H., Kim G., Han D.H., Lee M., Kim I., Kim B., Kim K.H., Song Y.M., Yoo J.E., Wang H.J. (2017). Ezetimibe ameliorates steatohepatitis via AMP activated protein kinase-TFEB-mediated activation of autophagy and NLRP3 inflammasome inhibition. Autophagy.

[237] Loomba R., Sanyal A.J., Kowdley K.V., Bhatt D.L., Alkhouri N., Frias J.P., Bedossa P., Harrison S.A., Lazas D., Barish R. (2023). Randomized, Controlled Trial of the FGF21 Analogue Pegozafermin in NASH. N. Engl. J. Med..

[238] Zeng X., Yang J., Hu O., Huang J., Ran L., Chen M., Zhang Y., Zhou X., Zhu J., Zhang Q. (2019). Dihydromyricetin Ameliorates Nonalcoholic Fatty Liver Disease by Improving Mitochondrial Respiratory Capacity and Redox Homeostasis Through Modulation of SIRT3 Signaling. Antioxid. Redox Signal..

[239] Xu X., Zhu X.P., Bai J.Y., Xia P., Li Y., Lu Y., Li X.Y., Gao X. (2019). Berberine alleviates nonalcoholic fatty liver induced by a high-fat diet in mice by activating SIRT3. FASEB J..

[240] Yan C., Zhang Y., Zhang X., Aa J., Wang G., Xie Y. (2018). Curcumin regulates endogenous and exogenous metabolism via Nrf2-FXR-LXR pathway in NAFLD mice. Biomed. Pharmacother..

[241] Wang X., Chang X., Zhan H., Zhang Q., Li C., Gao Q., Yang M., Luo Z., Li S., Sun Y. (2020). Curcumin and Baicalin ameliorate ethanol-induced liver oxidative damage via the Nrf2/HO-1 pathway. J. Food Biochem..

[242] Hammoutene A., Laouirem S., Albuquerque M., Colnot N., Brzustowski A., Valla D., Provost N., Delerive P., Paradis V., QUID-NASH Research Group (2023). A new NRF2 activator for the treatment of human metabolic dysfunction-associated fatty liver disease. JHEP Rep..

[243] Yue S., Zhou H., Wang X., Busuttil R.W., Kupiec-Weglinski J.W., Zhai Y. (2017). Prolonged Ischemia Triggers Necrotic Depletion of Tissue-Resident Macrophages To Facilitate Inflammatory Immune Activation in Liver Ischemia Reperfusion Injury. J. Immunol..

[244] Zhang Y.F., He W., Zhang C., Liu X.J., Lu Y., Wang H., Zhang Z.H., Chen X., Xu D.X. (2014). Role of receptor interacting protein (RIP)1 on apoptosis-inducing factor-mediated necroptosis during acetaminophen-evoked acute liver failure in mice. Toxicol. Lett..

[245] Majdi A., Aoudjehane L., Ratziu V., Islam T., Afonso M.B., Conti F., Mestiri T., Lagouge M., Foufelle F., Ballenghien F. (2020). Inhibition of receptor-interacting protein kinase 1 improves experimental non-alcoholic fatty liver disease. J. Hepatol..

[246] Qian X., Jin P., Fan K., Pei H., He Z., Du R., Cao C., Yang Y. (2024). Polystyrene microplastics exposure aggravates acute liver injury by promoting Kupffer cell pyroptosis. Int. Immunopharmacol..

[247] Coll R.C., Schroder K., Pelegrin P. (2022). NLRP3 and pyroptosis blockers for treating inflammatory diseases. Trends Pharmacol. Sci..

[248] Sun K., Wang J., Lan Z., Li L., Wang Y., Li A., Liu S., Li Y. (2020). Sleeve Gastroplasty Combined with the NLRP3 Inflammasome Inhibitor CY-09 Reduces Body Weight, Improves Insulin Resistance and Alleviates Hepatic Steatosis in Mouse Model. Obes. Surg..

[249] Mridha A.R., Wree A., Robertson A.A.B., Yeh M.M., Johnson C.D., Van Rooyen D.M., Haczeyni F., Teoh N.C., Savard C., Ioannou G.N. (2017). NLRP3 inflammasome blockade reduces liver inflammation and fibrosis in experimental NASH in mice. J. Hepatol..

[250] Marin-Aguilar F., Castejon-Vega B., Alcocer-Gomez E., Lendines-Cordero D., Cooper M.A., de la Cruz P., Andujar-Pulido E., Perez-Alegre M., Muntane J., Perez-Pulido A.J. (2020). NLRP3 Inflammasome Inhibition by MCC950 in Aged Mice Improves Health via Enhanced Autophagy and PPARalpha Activity. J. Gerontol. A Biol. Sci. Med. Sci..

[251] Yan S.L., Huang C.S., Mong M.C., Yin M.C. (2021). Oridonin Attenuates the Effects of Chronic Alcohol Consumption Inducing Oxidative, Glycative and Inflammatory Injury in the Mouse Liver. In Vivo.

[252] Liu D., Qin H., Yang B., Du B., Yun X. (2020). Oridonin ameliorates carbon tetrachloride-induced liver fibrosis in mice through inhibition of the NLRP3 inflammasome. Drug Dev. Res..

[253] Vergis N., Patel V., Bogdanowicz K., Czyzewska-Khan J., Fiorentino F., Day E., Cross M., Foster N., Lord E., Goldin R. (2021). IL-1 Signal Inhibition In Alcoholic Hepatitis (ISAIAH): A study protocol for a multicentre, randomised, placebo-controlled trial to explore the potential benefits of canakinumab in the treatment of alcoholic hepatitis. Trials.

[254] Jiang H., Zhang X., Yang W., Li M., Wang G., Luo Q. (2022). Ferrostatin-1 Ameliorates Liver Dysfunction via Reducing Iron in Thioacetamide-induced Acute Liver Injury in Mice. Front. Pharmacol..

[255] Tong J., Lan X.T., Zhang Z., Liu Y., Sun D.Y., Wang X.J., Ou-Yang S.X., Zhuang C.L., Shen F.M., Wang P. (2023). Ferroptosis inhibitor liproxstatin-1 alleviates metabolic dysfunction-associated fatty liver disease in mice: Potential involvement of PANoptosis. Acta Pharmacol. Sin..

[256] Li S., Zhuge A., Wang K., Xia J., Wang Q., Han S., Shen J., Li L. (2022). Obeticholic acid and ferrostatin-1 differentially ameliorate non-alcoholic steatohepatitis in AMLN diet-fed ob/ob mice. Front. Pharmacol..

[257] Wu A., Feng B., Yu J., Yan L., Che L., Zhuo Y., Luo Y., Yu B., Wu D., Chen D. (2021). Fibroblast growth factor 21 attenuates iron overload-induced liver injury and fibrosis by inhibiting ferroptosis. Redox Biol..

[258] Wang X., Sun K., Zhou Y., Wang H., Zhou Y., Liu S., Nie Y., Li Y. (2021). NLRP3 inflammasome inhibitor CY-09 reduces hepatic steatosis in experimental NAFLD mice. Biochem. Biophys. Res. Commun..

[259] Hu J.J., Liu X., Xia S., Zhang Z., Zhang Y., Zhao J., Ruan J., Luo X., Lou X., Bai Y. (2020). FDA-approved disulfiram inhibits pyroptosis by blocking gasdermin D pore formation. Nat. Immunol..

[260] Zhao J., Wang H., Zhang J., Ou F., Wang J., Liu T., Wu J. (2022). Disulfiram alleviates acute lung injury and related intestinal mucosal barrier impairment by targeting GSDMD-dependent pyroptosis. J. Inflamm..

[261] Cai Q., Sun Z., Xu S., Jiao X., Guo S., Li Y., Wu H., Yu X. (2022). Disulfiram ameliorates ischemia/reperfusion-induced acute kidney injury by suppressing the caspase-11-GSDMD pathway. Ren. Fail..

[262] Friedmann Angeli J.P., Schneider M., Proneth B., Tyurina Y.Y., Tyurin V.A., Hammond V.J., Herbach N., Aichler M., Walch A., Eggenhofer E. (2014). Inactivation of the ferroptosis regulator Gpx4 triggers acute renal failure in mice. Nat. Cell Biol..

[263] Mifflin L., Ofengeim D., Yuan J. (2020). Receptor-interacting protein kinase 1 (RIPK1) as a therapeutic target. Nat. Rev. Drug Discov..

[264] Oh J.H., Park S., Hong E., Choi M.A., Kwon Y.M., Park J.W., Lee A.H., Park G.R., Kim H.Y., Lee S.M. (2023). Novel Inhibitor of Mixed-Lineage Kinase Domain-Like Protein: The Antifibrotic Effects of a Necroptosis Antagonist. ACS Pharmacol. Transl. Sci..

[265] Zhu L., Chen D., Zhu Y., Pan T., Xia D., Cai T., Lin H., Lin J., Jin X., Wu F. (2021). GPX4-Regulated Ferroptosis Mediates S100-Induced Experimental Autoimmune Hepatitis Associated with the Nrf2/HO-1 Signaling Pathway. Oxid. Med. Cell Longev..

[266] Tucker B., Li H., Long X., Rye K.A., Ong K.L. (2019). Fibroblast growth factor 21 in non-alcoholic fatty liver disease. Metabolism.

